# Barriers to and facilitators of the implementation of Community Health Worker programmes in high-income countries: a systematic review

**DOI:** 10.1186/s12913-026-15114-9

**Published:** 2026-07-17

**Authors:** Andrew Riley, Hannah Doughty, James Edward Hill, Clarissa Giebel, Matthew Harris, Nefyn H. Williams

**Affiliations:** 1 Department of Primary Care and Mental Health, Institute of Population Health, University of Liverpool, Liverpool, L69 3GL UK; 2 School of Psychology, Research Centre for Brain and Behaviour, John Moores University, Liverpool, UK; 3 Synthesis, Economic Evaluation and Decision Science (SEEDS), University of Lancashire, Synthesis, Preston, UK; 4 Department of Primary Care and Mental Health, NIHR Applied Research Collaboration North West Coast, University of Liverpool, Liverpool, UK; 5 Department of Primary Care and Public Health, Imperial College London, London, UK; 6 Department of Primary Care and Mental Health, University of Liverpool, Liverpool, UK

**Keywords:** Community Health Workers, Implementation science, High-income countries, Systematic review, Health inequities, Workforce development, Primary health care, Framework synthesis, Social determinants of health

## Abstract

**Background:**

Global interest in Community Health Workers (CHWs) is rising as health systems seek to address persistent health inequities and the social determinants of health. However, in high-income countries (HICs), CHW implementation remains fragmented. This systematic review synthesises evidence on the factors that act as barriers and facilitators to the implementation and integration of CHW programmes across HIC primary healthcare settings.

**Methods:**

We searched MEDLINE, Scopus, PsycINFO, and CINAHL for studies published between January 2001 and August 2025. Inclusion focused on qualitative, quantitative, and mixed-methods papers regarding CHW implementation in HICs. Methodological quality was appraised using the Mixed Methods Appraisal Tool (MMAT). Data were synthesised using a ‘Best Fit’ Framework Synthesis and confidence in the findings was assessed via GRADE-CERQual.

**Results:**

Seventy-two papers were included (US *n* = 64; UK *n* = 4; Australia *n* = 3; Belgium *n* = 1), primarily comprising qualitative and mixed methods designs. Key facilitators included leadership buy-in, the presence of implementation champions, and established community partnerships that enabled resource sharing. Prominent barriers included precarious, short-term funding models that prioritised quantitative outputs over relational labour; hierarchical medical power dynamics; and disconnected IT systems that necessitated redundant data entry. The COVID-19 pandemic acted as a catalyst, elevating the CHW role within public health infrastructure while simultaneously exposing digital divides and structural fragility. GRADE-CERQual ratings indicated high confidence in findings regarding funding sustainability, training, and integration.

**Conclusions:**

The evidence synthesised in this review reveals a fundamental misalignment within current health services: while CHWs are valued for their unique relational capacity, they remain systemically undermined by transactional health system designs. Achieving sustainability requires a strategic shift toward long-term, flexible funding models and the workforce development of supportive infrastructure, including reflective supervision and standardised training. Integration must move beyond short-term, project-based initiatives toward a resilient and permanent public health workforce capable of addressing structural health inequities.

**Trial registration:**

PROSPERO: CRD42022310789.

**Supplementary Information:**

The online version contains supplementary material available at https://doi.org/10.1186/s12913-026-15114-9.

## Background

Global interest in large-scale Community Health Worker (CHW) programmes is growing as health systems seek to address persistent health inequities and the social determinants of health [1]. The term CHW encompasses a broad range of frontline public health roles that serve as a trusted liaison between formal health services and marginalised communities [2, 3]. CHWs are defined by the World Health Organization (WHO) and the American Public Health Association as lay public health professionals who are trusted members of, or possess an unusually close understanding of, the communities they serve [4, 5]. This trusted relationship is rooted in shared life experiences, languages, and socioeconomic backgrounds, as these workers are typically recruited directly from the populations they support [6].

CHWs play a critical role in the delivery of primary healthcare in low- and middle-income countries (LMICs) [7–11], where they operate under various titles, including community navigator, promotora, ASHA, lady health worker, and community health advisor [6]. Their engagement is considered a cost-effective strategy to address the projected 11 million global health worker shortage by 2030 [12, 13]. The CHW workforce has achieved substantial scale internationally and is active across multiple advanced economies. For example, the United States possesses a cadre of over 86,000 active CHWs employed across hospital, outpatient, and governmental sectors [14, 15]. Structured programmes also operate across other high-income country settings, including Canada [16], Australia [17], Belgium [18], and the UK [19] demonstrating that lay health work is an established and growing strategy within advanced health systems.

CHWs are deployed in high-income countries (HICs) to help address a range of complex and overlapping pressures from ageing populations, a rising prevalence of non-communicable chronic diseases, and deep-seated health inequalities [20–22]. Systematic reviews and meta-analyses of literature from high-income settings establish that CHW interventions are associated with measurable clinical improvements in chronic disease management [23], enhanced glycaemic control for individuals living with type 2 diabetes [24], increased uptake of preventative breast and cervical cancer screenings among historically non-adherent groups [25, 26], and improved health-related quality-of-life for individuals navigating complex, chronic conditions such as chronic obstructive pulmonary disease [27] and HIV [28].

CHW initiatives have been successfully deployed in high-income settings focusing on a wide range of health issues and marginalised populations, from bilingual promotoras, or Latinx CHWs, delivering environmental safety navigation in the United States [29], to community navigators supporting immigrant groups in Australia [30], and health trainers addressing lifestyle-related public health challenges in the United Kingdom [31]. However, implementation models for CHW programmes vary significantly by economic context. In advanced economies, CHW workforces are often integrated into health services as discrete, localised, or time-limited initiatives targeting specific geographic territories or populations, rather than operating as a uniform national infrastructure [21]. In many LMICs, such as Brazil and Ethiopia, CHWs are integrated as permanent and salaried pillars of nationalised primary care strategies designed to expand health access and achieve universal health coverage [32, 33] via formalised task shifting to mitigate scarcity in the professional clinical workforce [34, 35]. Conversely, in advanced economies, healthcare delivery is typically structured around highly professionalised, state-regulated clinical models and formal credentialing frameworks [5, 21]. The broad literature notes that within these heavily institutionalised settings, the deployment of non-clinical lay workers frequently introduces complex integration dynamics alongside licensed clinical professionals [36, 37].

Global public health bodies, including the WHO, have highlighted a dearth of evidence regarding the implementation of CHW programmes in advanced economies, calling for rigorous research to identify how contextual factors and regulatory environments impact the applicability of policy options [5, 38]. Moreover, because implementation success is highly contingent upon contemporary environmental factors, there is a critical need to examine how these structural boundaries persist or evolve over time, particularly following recent periods of health system stress like the COVID-19 pandemic [39, 40]. To address these concerns, this systematic review aims to provide the first comprehensive synthesis of international evidence on the factors that act as barriers and facilitators to the implementation and integration of community health worker programmes in high-income settings, establishing a necessary baseline for workforce deployment and long-term sustainability.

## Methods

This mixed-methods systematic review adheres to the Preferred Reporting Items for Systematic Reviews and Meta-Analyses (PRISMA) guidelines [41]. A mixed-methods approach was adopted to enable the quantification of reported barriers and facilitators alongside a qualitative exploration of how and why these factors operate within HIC health systems. The study protocol was registered in advance and amended to incorporate a refinement of the search strategy (PROSPERO: CRD42022310789).

### Search strategy

A comprehensive search strategy was developed in consultation with an information specialist to capture CHW implementation in HICs defined by OECD membership [42]. Four electronic databases (MEDLINE via Ovid, Scopus, PsycINFO, and CINAHL) were searched using a combination of indexed terms and free-text keywords. The search included studies published in English between 1 January 2001 and 5 August 2025. Eligible literature encompassed peer-reviewed journal articles, doctoral theses, and practice-based case studies. Formal grey literature database searches were not conducted, and evidence in languages other than English was not included, due to resource constraints. The lower time limit of 2001 was applied to enhance relevance to modern health service configurations rather than historical models operating within older policy environments, particularly following international initiatives to expand and formalise CHW workforces in the early 2000s [43, 44]. Search strategies are provided in Additional File 1. The database search was supplemented by a manual backward citation search of the reference lists of all included studies and relevant systematic reviews.

### Eligibility criteria

Qualitative, quantitative, and mixed-methods papers were eligible if they reported on factors influencing the implementation of CHW programmes within HICs. The review followed the Population, Concept, and Context (PCC) framework for study selection:

- Populations: Community Health Workers (CHWs) and key stakeholders involved in programme delivery, management, or integration, including healthcare providers, clinical staff, programme managers, administrators, and commissioners.
- Concept: Factors influencing the implementation or delivery of CHW interventions. Studies evaluating clinical or biomedical patient outcomes exclusively, without reporting data on implementation determinants, barriers, or facilitators, were excluded.
- Context: Community and primary care settings within high-income countries (OECD members).

Interventions delivered within inpatient hospital settings or educational institutions (schools) were excluded. While schools are widely recognised as community platforms within broad public health frameworks, they represent highly structured, institutional environments. To maintain conceptual consistency, this review restricted its scope to non-institutional, territory- or household-based community environments. This boundary was established to capture implementation determinants within general community and primary care infrastructures rather than within specialised institutional platforms. CHWs were defined in accordance with the American Public Health Association as lay individuals with a close understanding of their community, possessing job-related training shorter than that of a professional healthcare worker, whose primary aim is culturally appropriate health promotion [4].

### Study selection and quality appraisal

Two reviewers (AR and HD) independently screened titles and abstracts using Rayyan (Rayyan Systems Inc., Cambridge, MA) [45]. Country eligibility was assessed manually during screening as geographic contexts were readily apparent, while population exclusions primarily comprised non-lay healthcare roles and social prescribing link workers who fell outside the CHW definition. Full texts of potentially relevant articles were then independently assessed against the inclusion criteria by both reviewers. Disagreements at both stages were resolved through discussion or consultation with a third reviewer. Methodological quality was assessed using the Mixed Methods Appraisal Tool (MMAT) [46]. Because the sample included programmatic case studies and doctoral theses, papers were categorised based on the specific empirical data collection and analytical methods used to generate their findings (e.g., qualitative, quantitative, or mixed methods) rather than the overarching study label or publication format. The two reviewers independently appraised each study, following an initial calibration exercise conducted on 10% of the sample to ensure consistency. Any subsequent discrepancies in appraisal were resolved through consensus.

### Data extraction and synthesis

Data extraction and synthesis employed the ‘Best Fit’ Framework Synthesis (BFFS) approach [47, 48]. The conceptual framework developed by Kok et al. [49] was selected as a foundational model for this study. The widely cited model [5] was chosen because it theorises how the interaction between intervention design and contextual factors—spanning community, health system, and national levels—influences CHW performance. While originally based on evidence from LMICs, its comprehensive structure provided a robust starting point for adaptation to high-income settings. The framework was adapted a priori to exclude impact level factors. During synthesis, it evolved further to capture emerging themes, such as IT and Data Systems and the COVID-19 pandemic. The final adapted framework organised factors into four domains: Outer Setting, Health System, Intervention Design, and Individual Factors. A data extraction form incorporating the framework’s constructs was piloted, with data items including study characteristics, CHW roles, and contextual factors influencing CHW programme implementation. Data extraction was performed independently by one reviewer (AR) and checked by a second (HD). The full extraction sheet is provided in Additional File 2.

### Integration of quantitative evidence

A convergent integrated approach was adopted to combine different data types within a single synthesis [50]. Where retrieved quantitative data were highly descriptive or lacked the homogeneity required for statistical pooling, findings were “qualitised” by transforming numerical data, descriptive statistics, and survey results into narrative descriptive statements [51]. These transformed statements were then coded and synthesised inductively and deductively within the adapted framework alongside the primary qualitative data.

### Confidence assessment

Confidence in the findings was assessed using GRADE-CERQual, the standard for transparently evaluating how well review findings represent the phenomena of interest. One reviewer (AR) evaluated the findings against the four components—methodological limitations, coherence, adequacy of data, and relevance—to produce summary statements and an overall confidence rating (high, moderate, low, or very low) for each finding. These assessments were verified by a second reviewer (HD), with any discrepancies resolved through discussion and consensus.

### Manuscript preparation and LLM disclosure

During the preparation and drafting of this manuscript, the authors used an LLM (Gemini 3 Flash) to assist with structural organisation, formatting according to journal-specific guidelines, and proofreading for grammatical accuracy and clarity. All intellectual content, data synthesis, and conclusions were critically reviewed and verified by the authors, who maintain full accountability for the accuracy and integrity of the work.

## Results

### Study selection and appraisal

**Fig. 1:**
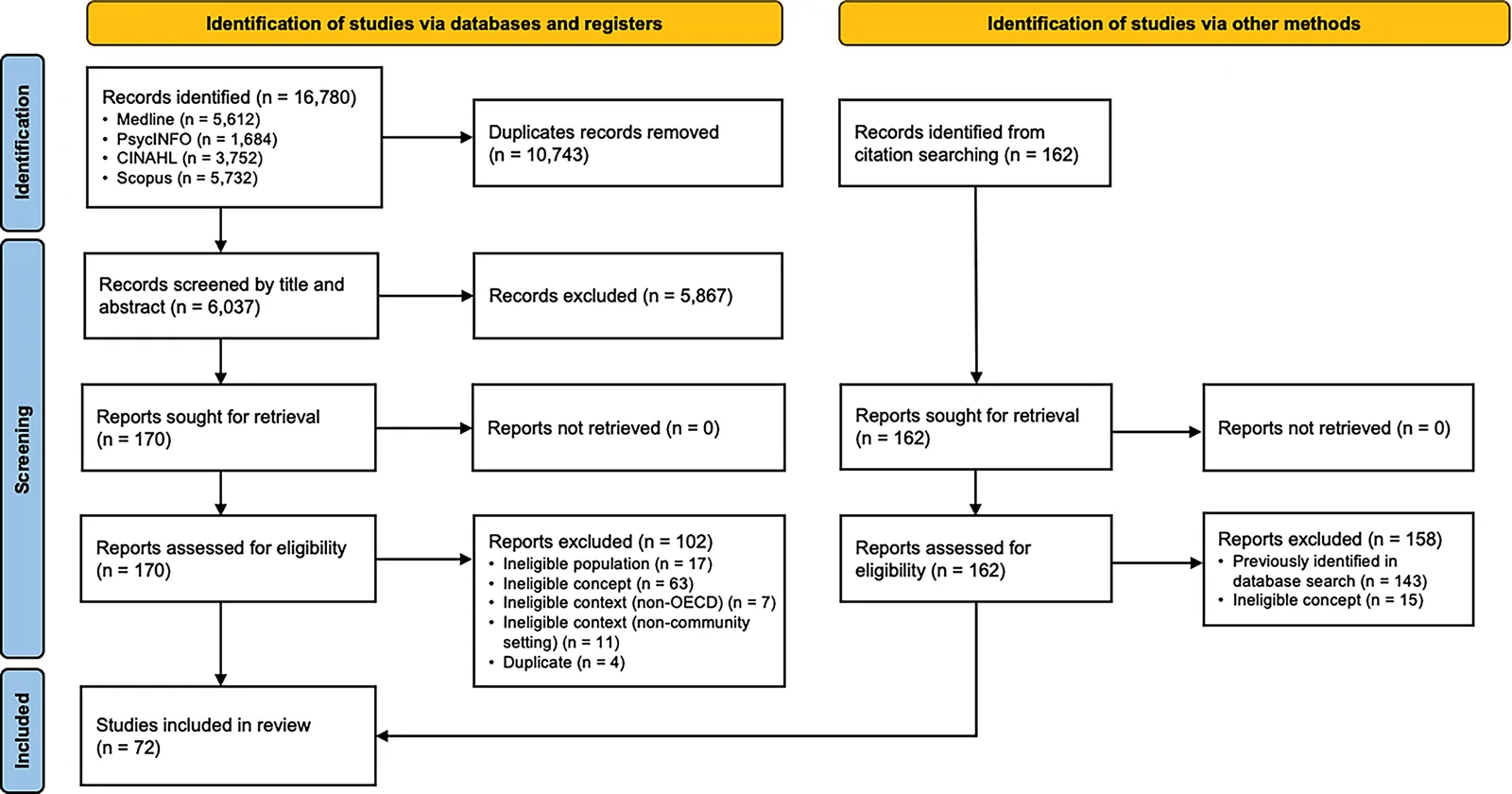
PRISMA 2020 flow diagram for new systematic reviews

Excluding duplicates, a total of 6,037 records were screened (Fig. 1). Following full-text assessment, 72 papers met the inclusion criteria [29, 52–122]. The search across MEDLINE, Scopus, PsycINFO, and CINAHL was supplemented by backward citation searching, which identified four additional studies. A total of 71 papers were appraised using the MMAT. One descriptive practice note that did not employ an empirical research design [60] was excluded from formal quality appraisal but retained for thematic synthesis due to its rich contextual data. Methodological appraisal found that the included evidence was of overall high quality. While most studies met all appraisal criteria, several papers had moderate limitations due to insufficient integration of qualitative and quantitative components [63, 72, 80] or a lack of methodological transparency in reporting [74, 81] (Additional File 1). These design-specific weaknesses, alongside issues of data adequacy and thematic coherence, directly informed the final GRADE-CERQual confidence ratings assigned to each review finding. Following the evidence synthesis, GRADE-CERQual assessments provided high confidence in four of the 18 summary findings and moderate confidence in the remaining 14 (Table 1; Additional File 1).

**Table 1:** Summary of qualitative themes and GRADE-CERQual assessment of confidence

Domain and theme	Summary of review finding	Studies contributing to the finding	GRADE-CERQual assessment of confidence	Explanation of GRADE-CERQual assessment
Outer setting factors				
Government and policy	Policy decisions were critical determinants of programme success; misalignment between policy design and need created structural barriers, while CHW collaboration in policy design facilitated implementation.	[52, 54, 56, 57, 59, 63, 66, 69, 73, 74, 77–79, 82, 84, 86, 87, 92, 102, 105, 107, 108, 110, 112–114, 120, 121]	Moderate confidence	Moderate concerns: coherence; adequacy Minor concerns: methodological limitations; relevance
Certification	CHW accreditation was valued for facilitating professionalisation but variation in its application was confusing and a focus on academic over lived experience hindered workforce diversity.	[54, 59, 60, 62, 66, 69, 73, 78–80, 86, 88, 91, 95, 101, 103, 108, 110, 112–115, 120, 121]	Moderate confidence	Moderate concerns: adequacy. Minor concerns: coherence
COVID-19	The pandemic exacerbated existing inequalities while simultaneously creating new barriers. The relational support provided by CHWs was critical for helping communities during the crisis.	[53, 54, 56–59, 61, 63, 66, 73, 76–79, 81, 84, 92, 93, 96, 102, 105, 107–110, 114, 120]	High confidence	Minor concerns: coherence; adequacy
Health system factors				
Funding and sustainability	Securing sustainable funding was a pervasive challenge. Short-term grants and restrictive reimbursement models were barriers to job security and often prioritised quantitative outputs over relational labour.	[52, 54, 56, 57, 59–61, 63, 66, 69–71, 73–76, 78, 79, 81–83, 85–87, 90–92, 100–102, 104, 105, 107–114, 116, 120, 122]	High confidence	Minor concerns: coherence, adequacy
Organisational readiness	Organisational cultures and professional norms facilitated readiness to adopt a CHW service. Successful integration required leadership buy-in combined with staff engagement to overcome hierarchical resistance.	[53, 54, 56, 57, 63, 66, 69, 70, 74, 75, 77–80, 84, 87, 89, 90, 92, 96, 102, 104, 105, 109, 110, 112, 116, 121]	Moderate confidence	Moderate concerns: coherence; adequacy
Intervention factors				
Intervention design	Healthcare initiations with clear scope of practice enabled CHWs to focus on key functions like outreach and relationship-building. Flexibility and cultural relevance were key facilitators, whereas rigid or non-translated materials hindered outreach.	[52, 54, 56, 57, 59, 60, 62–64, 67–70, 73, 75, 77, 79–81, 86, 92, 102, 108–110, 116, 117, 120–122]	Moderate confidence	Moderate concerns: coherence; adequacy
Recruitment	Proactive, community-based recruitment strategies were essential for identifying suitable candidates; however, traditional HR protocols often acted as a barrier by prioritising formal education over lived experience.	[29, 53, 54, 60–63, 69, 73, 75, 79, 81, 82, 87, 89, 91, 92, 96, 97, 101, 103, 105, 107, 108, 112, 114, 120]	Moderate confidence	Moderate concerns: adequacy
Training	Training enhanced skills and self-efficacy while facilitating integration. Effective training was practical, continuous, and covered both clinical topics and ‘soft skills’ like trauma-informed care.	[29, 52–55, 57, 59, 60, 62, 63, 66, 68–70, 72, 73, 75, 77–84, 86–92, 95, 97, 100–103, 108–114, 116, 120, 121]	High confidence	Minor concerns: coherence; adequacy
Incentives	Low wages were a pervasive threat to sustainability. Non-financial incentives were valued but insufficient to prevent turnover driven by financial precarity.	[29, 54, 57, 59, 60, 66, 69, 77, 78, 81–83, 86, 92, 100–102, 107, 108, 110, 112–114, 121]	Moderate confidence	Moderate concerns: adequacy Minor concerns: coherence
Supervision	Supervision that was responsive, understanding of the role, and developmental contributed to a resilient workforce; poor supervision was linked to worker turnover.	[52–55, 57, 59, 60, 62, 63, 66–70, 73, 74, 77–81, 83, 84, 91, 97, 100–103, 107–110, 112–118, 120]	Moderate confidence	Moderate concerns: coherence Minor concerns: methodology; adequacy
Communication and integration	Limited understanding of the CHW role among stakeholders acted as a barrier to integration. Clear role definitions, champions, and structured communication facilitated integration.	[52–57, 59, 60, 62, 66–73, 77, 78, 80, 81, 84, 86–88, 90, 92, 96, 97, 99, 101, 102, 104–107, 109–116, 118–122]	High confidence	Moderate concerns: coherence Minor concerns: adequacy
Quality assurance and evaluation	Service evaluation contributed to programme sustainability but was hindered by a mismatch between outcome metrics and the preventative, relational nature of CHW work.	[29, 52, 53, 57, 59, 60, 71, 79–81, 83, 84, 86, 87, 97, 102, 104, 107, 108, 110, 116, 118, 120]	Moderate confidence	Moderate concerns: adequacy Minor concerns: coherence
IT and data systems	IT systems facilitated integration when interoperable but acted as a barrier when workers lacked access or systems were not optimised for CHW use.	[52, 53, 55, 57, 60, 63, 69, 70, 73, 74, 77, 80–82, 87, 88, 90, 91, 93–97, 101, 102, 108, 110, 111, 116, 118, 120]	Moderate confidence	Moderate concerns: coherence; adequacy Minor concerns: relevance
Other resources	Resource constraints, particularly regarding physical space and client aid, hindered outreach. Collaborative partnerships were essential for supplementing gaps in service provision.	[29, 53, 54, 57, 59–61, 63, 70, 71, 75, 77, 79–84, 86, 88–90, 92, 93, 97, 99, 100, 102, 103, 107, 108, 111, 113, 114]	Moderate confidence	Moderate concerns: adequacy Minor concerns: relevance
Engaging clients	Trusted relationships were facilitated by shared experience and proactive outreach such as door-to-door canvassing and home visits	[52–55, 57–60, 62–65, 67–70, 73, 75–81, 84–86, 90, 92, 94–96, 98, 102, 105, 107–114, 116, 117, 119, 120]	Moderate confidence	Moderate concerns: adequacy Minor concerns: coherence
Engaging partner organisations	Community partnerships were key facilitators, improving outreach, reducing duplication, and enabling bi-directional resource sharing. However, bureaucratic hurdles could strain these relationships.	[52–54, 56–60, 63, 67–69, 73–75, 77, 79–81, 83, 84, 86, 88–90, 92, 96, 98, 99, 102, 107, 108, 110–114, 116, 118, 120–122]	Moderate confidence	Serious concerns: adequacy Moderate concerns: coherence
Individual factors				
CHW motivation and self-efficacy	CHWs were intrinsically motivated by a desire to help their communities and valued professional development. However, burnout from vicarious trauma, low pay, and a lack of recognition threatened retention.	[29, 52–57, 59, 60, 62, 64, 66–70, 73, 76–81, 83, 84, 86, 88, 90, 92, 93, 95, 102, 103, 107, 108, 110, 112–117, 120]	Moderate confidence	Moderate concerns: adequacy; coherence
Service users	Access was hindered by structural barriers including poverty, transport, and systemic marginalisation. Trust in CHWs helped navigate these barriers, but external constraints often limited engagement capability.	[53, 60, 62–70, 73, 74, 77–79, 84–89, 94, 96, 102, 103, 107, 109, 111, 114–116, 122]	Moderate confidence	Moderate concerns: adequacy Minor concerns: coherence; relevance

### Evidence overview

The included evidence consisted of 51 qualitative studies [29, 52, 54, 56–59, 61, 62, 64–67, 69–71, 74–79, 83–86, 88, 89, 91–99, 101, 104, 106, 107, 109, 111, 113, 115–117, 119–122], 17 mixed methods papers [53, 55, 63, 68, 72, 73, 80, 81, 87, 90, 100, 102, 103, 110, 112, 114, 118], three quantitative studies [82, 105, 108], and one descriptive practice note [60]. Included in this total were two doctoral theses [54, 81]. Most studies were conducted in the US (*n* = 64) [29, 52–55, 57–63, 66, 68–72, 74–86, 88–115, 117–121]. Other settings included the UK (*n* = 4) [65, 73, 87, 116], Australia (*n* = 3) [64, 67, 122], and Belgium (*n* = 1) [56]. Most studies (*n* = 48) defined the CHW role with reference to the American Public Health Association, characterising them as frontline public health workers possessing close understanding of the communities they serve [52, 54–56, 58, 59, 61, 62, 66, 69–72, 74, 76–78, 81, 82, 85, 86, 88–93, 95, 97, 98, 100, 101, 103–115, 118–121]. Alternative CHW titles included ‘lay health worker’ [29, 67], ‘promotora’ [57, 60, 63, 75, 79, 83, 94, 99, 102, 117], ‘COVID-19 health ambassador’ [53], ‘health navigator’ [84] ‘health trainer’ [65, 87], ‘indigenous health worker’ [64], ‘community health advocate’ [96], ‘community health representative’ [80], ‘link worker’ [122], and ‘community health and wellbeing worker’ [116]. Across all settings, key functions undertaken by the CHWs included service navigation, health promotion and education, addressing vaccine hesitancy, and assisting clients in meeting unmet social needs.

Some papers provided granular detail of the CHW role within workplace and community contexts, including primary care practices and community clinics [54, 61, 62, 65, 68–70, 72, 77, 80, 81, 85, 87, 89, 90, 96, 99, 103–106, 109, 112–114, 116, 118, 121], community-based organisations (CBOs) [29, 52, 55, 57–59, 63, 66, 77, 81, 83, 85, 92, 98, 100, 102, 107, 110, 112, 114, 121], client homes [68, 79, 80, 91, 99, 106, 117], and health departments [29, 52, 69, 77, 78, 82, 110, 112, 114, 121]. Others described interventions from a systems or policy perspective, illustrating high-level factors influencing programme implementation at the macro or state level [69, 71, 74, 82, 86, 87, 92, 101, 103, 108, 110, 112, 115, 120, 121]. Others focussed on CHW interventions characterised by particular aspects of the CHWs’ background, such as having lived experience of the criminal justice system [77], being former refugees [84], or being members of a particular ethnic group e.g., the Navajo Nation [80]. The majority of studies provided evidence through first-hand testimony from stakeholders directly involved in their delivery or commissioning, including CHWs [52–56, 58, 59, 62, 64–66, 68, 70, 73, 74, 76–79, 81, 82, 84, 85, 88, 90, 93–96, 98–100, 102–104, 107–120], supervisors and administrators [29, 52, 59, 69–71, 77, 78, 83, 88, 89, 91, 92, 96, 100, 101, 108–110, 112, 120, 122], clinicians [61, 68, 70, 72, 89, 96, 104–106, 109, 113, 114, 118], and service users [53, 62, 68, 73, 100, 109, 122].

### Qualitative evidence synthesis

The following section presents the qualitative synthesis, organised by the study’s four overarching domains (Outer Setting, Health System, Intervention Design, and Individual Factors), followed by implementation themes and GRADE-CERQual summary statement. Table 1 identifies the studies relevant to each theme, alongside GRADE‐CERQual confidence assessments, while Table 2 provides illustrative quotes.

**Table 2:** Selected illustrative quotes

Domain & Theme	Source & Context	Illustrative Quote
**Outer Setting Factors**		
Government and Policy	Stupplebeen et al., 2019 (US)	“I think that’s where we need to move is towards statewide policies that really support the CHW … We need something at the legislative level that would support that as a legitimate position and role, that would actually get paid.” CHW programme administrator
Certification	Mason et al., 2011 (US)	“There is an inherent tension between promoting heightened professional status for CHWs and retaining the very characteristics that make CHWs distinctive and effective—their ties and orientation to communities they serve.”
**Inner Setting Factors**		
Funding & Sustainability	Smithwick et al., 2023. (US)	“You know how it works when you work with grants, you have this person for 1 year … she’s gained so many skills, and then she has to leave because I don’t have any more funds.” CHW Manager
Organisational Readiness	Fuller et al., 2024 (US)	“I have no problems telling you that we were leery about [bringing on the CHW] … bringing someone in that we didn’t have a say so in who they picked or what this background was.” Clinic Leader
**Intervention Factors**		
Programme design	Gunter et al., 2023 (US)	To ensure services aligned with regulations, the programme “emphasized CHW visits include health education, self-management skill building, and resource connections.”
Recruitment	Ingram et al., 2025 (US)	“The recruitment and hiring protocols favor people with education… and the scoring rubrics make it difficult to recognize soft skills such as building rapport”
Training	Kirimi, 2024	“Motivational Interviewing was a serious game changer [because] being able to effectively communicate … is a must”
Incentives	Rahman et al., 2021 (US)	“They can go to McDonald’s and make 15 bucks an hour and they’re going to come and do this really hard work for $12?” Programme Director
Supervision	Wilkinson et al., 2016 (US)	“My supervisor and my manager, they actually started out with me as working in the field … they’re very understanding.” CHW
Communication and integration	Tredinnick-Rowe et al., 2024 (UK)	“Interviewees described the seamless connection and interaction between the CHWs and other members of the team, as well as with other organizations providing services in their community.”
	Allen et al., 2015 (US)	“I feel like my voice is heard but … it’s like I’m a little fish in a big pond because there are so many other things that they’re focusing on right now, that sometimes my role and position gets put on the back burner.” CHW
Other resources	Lapidos et al., 2021 (US)	“You offer this service … They feel like … ‘I’m going to get help!’ and then we have to say, ‘Never mind, you’re not going to get it’” CHW
Engaging clients	Sherman et al., 2017 (US)	“You know, you have to develop that trust. And people will know if you’re being fake.”
**Individual Factors**		
CHW motivation and self-efficacy	Kirimi, 2024 (US)	“I know this is a calling for me … this is much greater than a paycheck.” CHW
Service users	Carson et al., 2022 (US)	“They’ll say, ‘well, so-and-so said they were going to help me, but it never happened.’ … why should I trust you?” CHW

### Outer setting factors

#### Government and policy

**Finding 1: Policy decisions were critical determinants of programme success; misalignment between policy design and need created structural barriers**,** while CHW collaboration in policy design facilitated implementation.**

Policy-level decisions were identified by healthcare planners and commissioners as powerful, overarching determinants of implementation success. Policy misalignment proved highly detrimental: the UK’s Health Trainer initiative was impeded by a fundamental disparity between the intervention design (developed via government policy paper) and actual population need [87]. Furthermore, a mid-course policy change enabling GPs to redeploy trainers to activities beyond their scope undermined fidelity and retention [87]. Conversely, successful implementation foregrounded “authentic collaboration,” where CHWs acted as architects of policy rather than mere subjects, effectively leveraging policy windows to secure recognition, a dynamic documented in a narrative case study of state-level advocacy campaigns involving CHW leaders and public health officials [86]. However, restrictive funding mechanisms frequently acted as barriers. Gunter et al. [74] found from evaluations capturing both technical assistance staff and CHWs that Medicaid policies reimbursing only for narrow “health education” failed to cover the holistic support CHWs provide. Structural misalignment could also force organisations to piece together fragmented grants, fostering job insecurity, an issue raised by regional CHW leaders [110]. Furthermore, policies could actively exclude target populations: Sainz et al. [107] noted in a study of 15 Latinx CHWs working within a community-based organisation that regulations excluding undocumented individuals from aid created critical barriers that CHWs had to navigate to support their clients. This was reinforced by an evaluation across five Medicaid Managed Care Organisations (MCOs), where MCO administrators, supervisors, and CHWs reported that rigid corporate billing metrics restricted holistic care workflows [120].

### Certification

**Finding 2: CHW accreditation was valued for facilitating professionalisation but variation in its application was confusing and a focus on academic over lived experience hindered workforce diversity.**

The credentialing process, available to CHWs in the US, was valued by programme administrators and CHWs for legitimising the workforce, standardising skills, and creating career pathways [54, 88, 95, 101, 103]. For highly integrated cadres, such as a cohort of 107 bilingual Navajo Nation Community Health Representatives who are required to hold Certified Nursing Assistant qualifications, certification was essential for formal clinical team alignment [80]. Leaders also considered it essential for accessing reimbursement streams [74]. However, certification mandates could act as a barrier to recruitment when institutional protocols favoured formal education over “soft skills” or lived experience [78]. Based on interviews with 60 CHWs and supervisors within community-based organisations, Billimek et al. [59] warned that models prioritising didactic training threatened the core CHW identity by excluding those with the necessary “connective capacity.” To mitigate this, some programmes implemented tiered systems where experience provided a pathway for advancement alongside certification [78]. Additionally, inconsistent state-level policies and a confusing multiplicity of accreditation programmes impacted workforce planning, causing systemic obstacles for executive directors and frontline workers alike [95, 101, 103, 115], while the cost of certification was also a major barrier, with leaders reporting that it limited recruitment and threatened programme sustainability [91, 103, 109].

### COVID-19

**Finding 3: The pandemic exacerbated existing inequalities while simultaneously creating new barriers. The relational support provided by CHWs was critical for helping communities during the crisis.**

The COVID-19 pandemic acted as both a stressor and a catalyst across diverse academic, healthcare, and community alliances [58, 61, 73, 76, 93, 96, 109]. It introduced barriers such as the “digital divide,” which hindered virtual outreach, according to qualitative data captured across 11 academic sites and over 75 community partners [57], and required CHWs to combat disinformation [92]. Government-led responses, such as vaccine mandates or military presence at clinics, occasionally triggered trauma and withdrawal among specific populations, requiring CHWs to act as mediators [84]. Despite these challenges, the crisis underscored the CHW role as “trusted messengers” to investigators and community representatives alike [58, 63, 76]. With the closure of other support agencies, CHWs became a critical source of in-person relational support [58, 73, 76, 96, 107], confirming their function as a frontline service adaptable to systemic failures, a perspective shared by focus groups of 21 CHWs embedded within grassroots community-based organisations [66].

### Health system factors

#### Funding and sustainability

**Finding 4: Securing sustainable funding was a pervasive challenge. Short-term grants and restrictive reimbursement models were barriers to job security and often prioritised quantitative outputs over relational labour.**

A lack of sustainable financing served as a primary barrier to securing permanent employment contracts and maintaining stable workforce retention reported across studies surveying healthcare planners, managers, and frontline CHWs [61, 71, 83, 87, 91, 100, 101, 104, 109, 111, 114]. The reliance on short-term, fragmented grants created a cycle of job insecurity, causing high turnover as staff sought more stable employment [69, 101, 110]. The empirical scale of this risk was validated by a national survey of 1,858 CHWs, which identified dissatisfaction with organizational compensation and support as the single strongest predictor of intent to leave the public health workforce [82]. A structural mismatch was identified by executive directors and supervisors between funding metrics and CHW realities; funding often prioritised “products” (quantitative outputs) over “people” (relational trust), leading to abrupt contract terminations that undermined longitudinal care and community trust, as illustrated in a community-based participatory case study of 60 CHWs [59]. Furthermore, reimbursement rates in insurance-based systems frequently failed to cover the actual costs of service delivery or essential non-billable activities such as travel and care coordination [74]. Bureaucratic delays in distributing funds to community partners further threatened the operational viability of smaller organisations, according to multi-site academic and community alliance representatives [57]. Clinicians also recognised this barrier: a national survey of 1,504 US primary care providers found that 45% identified “lack of reimbursement” as the primary obstacle to integrating CHWs within primary care teams [105].

### Organisational readiness

**Finding 5: Organisational cultures and professional norms facilitated readiness to adopt a CHW service. Successful integration required leadership buy-in combined with staff engagement to overcome hierarchical resistance.**

Receptivity to CHW programmes was a critical determinant of success [75, 77, 87, 89, 96, 104, 109]. While strong leadership and the presence of “implementation champions” facilitated acceptance among a mixed-methods evaluation of clinicians, patients, and CHWs [68], leadership buy-in alone was insufficient if implementation was perceived as “top-down.” Such cultures were reported by clinic staff to limit innovation [104], whereas a collaborative culture among coordinated care organisation leaders [71] and a care philosophy aligned with social determinants of health reported by health system administrators and clinicians were associated with success [89]. Clinics assigned CHWs without involvement in the hiring process often reacted with wariness, as explicitly captured in a qualitative study of 14 clinic and project leaders [69]. Hierarchical power dynamics also inhibited integration; CHWs reported difficulties in making requests of clinicians across multiple ambulatory clinics [70], while health department supervisors and managers displayed scepticism regarding the capabilities of non-clinical workers [78]. To enhance readiness and integration, the suitability of a host organisation for adopting a CHW programme could be assessed against criteria like shared goals before implementation began, a process successfully piloted by non-profit health plan administrators [96].

### Intervention factors

#### Intervention design

**Finding 6: Healthcare initiations with clear scope of practice enabled CHWs to focus on key functions like outreach and relationship-building. Flexibility and cultural relevance were key facilitators**,** whereas rigid or non-translated materials hindered outreach.**

Interventions with clearly defined CHW roles, objectives, and modes of engaging communities, such as employing health education frameworks, self-management skill building, and directly linking clients to social welfare services, material aid, and local support networks, were viewed positively by evaluation teams and community partners [64, 67, 73, 117, 122]. With clearly defined objectives and tools at their disposal, Indigenous Health Workers delivering the Flinders Chronic Condition Management Program found it easier to focus on building relationships [64]. Programmes that gave promotoras conducting home visits flexibility to organise their own schedules [117], or allowed Lay Health Workers in Melbourne to determine the areas on which they wished to focus their efforts through an “empowerment model” [67], were felt to increase CHW self-efficacy and improve delivery. Culturally grounded innovations significantly aided engagement: for example, Johnson et al. [79] described how promotoras iteratively refined a nutrition curriculum for border communities, modifying recipes to ensure ingredients were affordable and culturally preferred. Conversely, interventions could impede CHWs when materials were not adapted for specific populations, such as undocumented patients or non-English speakers, an operational challenge observed across regional coalitions and nationwide clinical models [102, 109].

### Recruitment

**Finding 7: Proactive**,** community-based recruitment strategies were essential for identifying suitable candidates; however**,** traditional HR protocols often acted as a barrier by prioritising formal education over lived experience.**

Effective recruitment prioritised shared cultural backgrounds and interpersonal skills, according to programme coordinators and planners [29, 87, 91, 103]. Successful strategies included local recruitment drives, utilising plain-language job adverts posted in settings like schools and supermarkets [29, 97, 103], and leveraging partner networks across voluntary and community sector organisations [53, 73, 89, 101, 103]. Difficulties arose in finding candidates with the right mix of skills and qualities [91], particularly in rural areas. A major barrier emerged from institutional human resources (HR) protocols. Standardised scoring rubrics often favoured formal education and failed to recognise the “soft skills” or lived experience critical to the CHW role, as highlighted by health department managers [78]. This “bureaucratic hiring” barrier prevented the employment of qualified community members [92], forcing some programmes to create tiered entry systems to bypass educational requirements [78]. Some programme administrators talked about “low barrier hiring processes” and noted that lengthy online application forms were a primary obstacle to employing a less traditional healthcare workforce [89].

#### Training

**Finding 8: Training enhanced skills and self-efficacy while facilitating integration. Effective training was practical**,** continuous**,** and covered both clinical topics and ‘soft skills’ like trauma-informed care.**

Training was consistently linked to improved confidence, retention, and team integration [68, 72, 88, 97]. Core curricula typically covered public health, disease-specific knowledge, and cultural competence across primary care clinics and programmatic evaluations [55, 72, 89], such as the Texas C-STEP framework which trained over 33 CHWs [60]. Flexible and practical methods like continuous, on-the-job, and interactive training were preferred by site leaders and CHWs [29, 89, 100, 103, 109, 111], although there could be a trade-off between convenient online modalities and face-to-face sessions valued for peer support, as observed by research and voluntary sector teams [73, 89, 103]. Key challenges involved tailoring training to CHWs’ varied abilities, like digital literacy, and personal circumstances [87, 91, 101]. Studies highlighted the critical need for training in “soft skills,” particularly motivational interviewing, which a qualitative focus group of 12 CHWs identified as a major asset for addressing social needs [81], and trauma-informed care [66], to manage complex client needs. This was supported by a secondary mixed-methods trial analysis involving 12 CHWs and 17 primary care providers, which focused heavily on training for internal team integration boundaries [68]. A lack of training for other staff regarding the CHW role was identified as a significant gap that perpetuated role confusion among clinic leaders [69].

### Incentives

**Finding 9: Low wages were a pervasive threat to sustainability. Non-financial incentives were valued but insufficient to prevent turnover driven by financial precarity.**

Poor compensation was described as an endemic problem driving staff turnover and threatening sustainability across community-based organisations and public health departments [29, 83, 100, 101, 112]. A mixed-methods national evaluation incorporating a survey of 867 CHWs revealed that CHWs in some settings reported being “one paycheck away” from needing the services they provided, while highlighting payment delays within CBO networks [112]. While non-financial incentives—such as recognition, reimbursement for expenses, and access to professional networks—were appreciated by programme planners [29, 83], they did not fully mitigate the stress of low pay. An exploratory focus group study indicated that while “impact” and “purpose” were primary intrinsic motivators, the lack of pay parity with other roles was a source of frustration [81], while the reliance on volunteer CHWs, alongside paid, was viewed as unsustainable [92]. Statistical evidence reinforced this link between compensation and retention: Kirkland et al. [82] found in a quantitative survey of 1,858 public health agency CHWs that CHWs dissatisfied with their pay had a 39% probability of intending to leave their jobs.

#### Supervision

**Finding 10: Supervision that was responsive**,** understanding of the role**,** and developmental contributed to a resilient workforce; poor supervision was linked to worker turnover.**

Supportive supervision was identified as a key facilitator for wellbeing and self-efficacy by cadres working across health systems and non-profits [55, 67, 100, 103, 109, 115, 117]. Effective supervisors were described by urban and rural CHWs as relatable, responsive, and capable of providing developmental support rather than just administrative oversight [81, 103, 115]. Programme leaders suggested that supervision focus on building individual capacity [91, 97, 101, 118], while a “trauma-informed” approach, sensitive to the CHWs’ lived experience, was thought by executive directors to mitigate burnout and turnover [101]. CHWs specifically valued supervisors who understood the “real life” nature of community work, which often occurs outside standard business hours, especially those supervisors who had previously worked in the field themselves [81]. Peer-to-peer supervision and weekly debriefs were particularly valued for managing the emotional burden of the work, as highlighted by vaccine outreach health navigators and Melbourne-based Lay Health Workers [55, 67, 84, 113, 115]. However, a lack of supervisor capacity or understanding of the CHW role often left workers feeling unsupported, an issue raised within a municipal health justice network employing 18 CHWs with personal histories of incarceration [77]. Insufficient support was also a critical driver of attrition: Kirkland et al. [82] identified dissatisfaction with “organisational support” within their large quantitative dataset as the single strongest predictor of a CHW’s intent to leave the workforce.

#### Communication and integration

**Finding 11: Limited understanding of the CHW role among stakeholders acted as a barrier to integration. Clear role definitions**,** champions**,** and structured communication facilitated integration.**

Communication and role clarity were critical themes, with confusion about the CHW role among clinical colleagues and other key stakeholders impeding integration. Rohan et al. [105] found in a national survey of 1,504 primary care practitioners that one-third of primary care providers reported a “lack of clarity” about CHW functions as a major barrier. One supervisor noted in an evaluation of community-based and clinical initiatives that the CHW’s “biggest barrier is having the health care team understand what their role is” [111]. A source of this barrier was poor initial communication about the aims of the CHW service [71, 96, 111], which could cause unrealistic expectations about what CHWs could achieve among coordinated care organisation leaders [71] and fear of the CHWs encroaching on other staff roles, reported by preceptors and administrators [88]. In clinical settings, “hierarchical power dynamics” inhibited communication, as noted by ambulatory care clinicians and programme staff [70], while CHWs in some systems reported feeling “integral but not integrated” and “completely invisible” to the clinical team [90]. To enhance understanding and integration, proactive communication strategies were essential [96, 97]. These included formal onboarding processes where CHWs could explain their work to office staff and providers [88, 96, 99, 111] and purposeful integration into existing team communication channels [72]. Implementation champions were vital for raising buy-in [68, 96, 97, 99, 104, 118], while structured communication mechanisms, such as “huddles” [74] or bi-directional learning collaboratives comprising clinic and health department leaders [69], facilitated rapport. Without structured opportunities for clinicians to learn about the CHW role and develop connections with individual workers, and vice versa, CHWs felt isolated and clinical partners disconnected [70].

#### Quality assurance and evaluation

**Finding 12: Service evaluation contributed to programme sustainability but was hindered by a mismatch between outcome metrics and the preventative**,** relational nature of CHW work.**

Service evaluation was crucial for assessing CHW performance and justifying programme funding [29, 83, 87, 97, 104, 118], but faced significant challenges. Some CHWs felt there was a high documentation burden, which could impede relationship-building, with obesity-related programme planners across 10 states noting that extensive demands were often outside the core strengths of the workforce [83, 118]. In terms of measuring programme impact, some leaders noted a fundamental mismatch between outcome metrics mandated by funders and commissioners—often focused on throughput or short-term clinical outcomes—and the preventative, relational nature of CHW work [71, 87]. For example, Wennerstrom et al. [120] found that MCOs prioritised metrics like hospital readmission rates and “successful contact,” which administrators admitted failed to capture the full impact of the CHW’s work. Billimek et al. [59] highlighted that such models focus on “units of service” and miss the unseen labour of trust-building.

### IT and data systems

**Finding 13: IT systems facilitated integration when interoperable but acted as a barrier when workers lacked access or systems were not optimised for CHW use.**

IT tools had a mixed impact on programme implementation. Access to Electronic Health Records (EHRs) supported integration [55, 96, 97, 118], with some CHWs reporting that EHR access improved communication with providers [80]. However, onerous data entry could reduce the number of clients seen [95], and providers expressed liability concerns over EHR access for non-clinical employees [88]. There were also time and training costs, for CHWs who may not be familiar with computers, an obstacle observed by supervisors and executive directors [91, 101] and for existing staff required to work in new and complementary ways [97]. Technical barriers were also prominent. Acharya et al. [52] described from a feedback survey evaluating user entries how a lack of interoperability between CHW databases and EHRs created a “double documentation” issue. Tredinnick-Rowe et al. [116] highlighted in a qualitative evaluation of 10 frontline workers and 6 managers in Cornwall, UK, that restricted access to NHS systems forced CHWs to rely on detached spreadsheets, limiting their ability to share intelligence with clinical partners. Conversely, systems designed with CHW input were found to improve usability and client engagement [52].

### Other resources

**Finding 14: Resource constraints**,** particularly regarding physical space and client aid**,** hindered outreach. Collaborative partnerships were essential for supplementing gaps in service provision.**

A lack of material resources was a widespread concern across clinical and community-based sectors [29, 71, 83, 88, 89, 93, 97, 100, 103, 111]. Lack of dedicated physical space in clinics risked isolating CHWs from teams, leaving workers physically separated from physicians and underutilised by care teams [89, 97, 99, 111]. Many CHWs also often lacked resources to address clients’ basic needs, leading to frustration and feelings of disillusionment among frontline staff when they could not offer tangible help [81, 90]. To address this, successful programmes leveraged collaborative multi-agency partnerships between CBOs, universities, and health centres to provide wraparound services, such as food boxes or rent assistance [83, 102].

### Engaging clients

**Finding 15: Trusted relationships were facilitated by shared experience and proactive outreach such as door-to-door canvassing and home visits**.

The capacity to build trusted relationships was consistently cited as a facilitator for community engagement [64, 67, 76, 85, 89, 91, 93, 96, 101, 111, 122]. Critical elements of trust-building included “listening, consistency, investing time, and following through on commitments” reported by clinic administrators [89], as well as being transparent and showing genuine interest to priority populations experiencing deep-seated mistrust of statutory services [98]. It also stemmed from cultural competence and shared experience, providing a deep understanding of client challenges [58, 64, 76, 85, 94, 98, 109, 111]. Billimek et al. [59] defined this as “connective capacity”—a trust earned by being “present, visible, and available.” Effective engagement often required “high-touch” strategies such as door-to-door canvassing [57] and “repeated dialogue,” which a qualitative study of 6 refugee health navigators noted was vital to address vaccine hesitancy [84]. Home visiting was highlighted as a particularly powerful tool, described by one programme manager as “the single most important thing that happens” [91] because it allowed CHWs to observe family dynamics and other issues directly [85, 119]. Navigating family dynamics was also crucial; Johnson et al. [79] found in a father-focused nutrition initiative that female CHWs successfully recruited hard-to-reach fathers by first building rapport with mothers as the “gatekeepers” of the family. Outreach was effective when workers used local communication channels, like notice boards [58, 85, 111, 115], and had visual markers like uniforms [65].

### Engaging partner organisations

**Finding 16: Community partnerships were key facilitators**,** improving outreach**,** reducing duplication**,** and enabling bi-directional resource sharing. However**,** bureaucratic hurdles could strain these relationships.**

Collaboration with community partners was a core CHW function, strengthening referral networks and reducing duplication [37, 58, 91, 111, 122]. Casillas et al. [63] described from progress reports and surveys within a statewide alliance how statewide alliances allowed for “bi-directional information” sharing, enabling partners to repurpose materials and solve problems jointly. Pre-existing relationships were often the cornerstone of rapid implementation during the COVID-19 crisis [63, 92]. However, bureaucratic barriers, such as complex subcontracting processes required by academic or federal partners, could delay funding and strain relationships with smaller CBOs, affecting field models deploying hundreds of frontline promotoras [102]. Key mechanisms for building community partnerships included CHWs attending meetings and delivering presentations at partner organisations, sharing client intelligence, and co-creating local events [58, 91, 99, 111].

### Individual factors

#### CHW motivation and self-efficacy

**Finding 17: CHWs were intrinsically motivated by a desire to help their communities and valued professional development. However**,** burnout from vicarious trauma**,** low pay**,** and a lack of recognition threatened retention.**

CHWs were regularly described as intrinsically motivated by a desire to give back and challenge inequality [54, 67, 73, 88, 103]. Kirimi [81] found via an exploratory mixed-methods design that “impact” and “purpose” were top motivators for CHWs, often outweighing financial factors. However, Melbourne-based Lay Health Workers noted that low self-confidence could act as a barrier to executing responsibilities [67], while promotoras identified gender power imbalances when conducting outreach with male populations [94]. The emotional toll of working in under-resourced services and regularly being exposed to client trauma could lead to “compassion fatigue” [107] and burnout [64, 66, 76, 93, 95, 103, 115]. During the pandemic, CHWs were found to deprioritise their own wellbeing [59], necessitating robust mental health support systems to protect frontline staff within community-based frameworks [66]. These challenges led to increased worker turnover, creating barriers to programme sustainability [69]. Where CHWs received effective supervision and training [68], and received strong peer and clinical support [77], their confidence and resilience were enhanced.

#### Service users

**Finding 18: Access was hindered by structural barriers including poverty**,** transport**,** and systemic marginalisation. Trust in CHWs helped navigate these barriers**,** but external constraints often limited engagement capability.**

In terms of direct perspectives, service users directly participated in only three of the included studies, where they valued CHWs who were consistent and non-judgemental [73, 109, 114, 122]. However, the majority of evidence regarding client barriers was reported by CHWs and service providers, who observed that client engagement with CHW programmes was heavily constrained by external factors such as housing insecurity, lack of transport, and technical barriers [64, 93, 94, 103, 111] Systemic marginalisation [94, 115], including fear of immigration enforcement noted across more than 75 community partners [57] and medical mistrust rooted in historical injustice, recorded in a community intervention survey of 152 older African American adults [53], were also significant barriers. CHWs were uniquely positioned to bridge these gaps through their lived experience, such as a municipal network of 18 CHWs possessing personal histories of incarceration [77], and through the provision of home visits and accessible outreach delivered in community settings [67, 89, 91, 96]. However, for some populations, such as those defined within Indigenous health and justice-involved evaluations, competing priorities like obtaining vital documents or housing often took precedence over health promotion goals [64, 77, 87].

## Discussion

### Summary of main findings

This systematic review synthesised evidence from 72 studies to identify the determinants of CHW programme implementation in HICs. The findings highlight a fundamental tension: while CHWs are valued for their unique ability to bridge the gap between communities and formal health systems, the systems themselves are often structurally ill-equipped to support this relational work. At the outer setting, policy frameworks frequently failed to align reimbursement mechanisms with the holistic nature of CHW practice. The COVID-19 pandemic acted as a catalyst, elevating the CHW role to essential infrastructure while simultaneously exposing digital and structural barriers. Within health systems, the review identified a people-versus-product tension, where funding models prioritised short-term clinical outputs over the long-term relational trust-building central to the CHW identity. Implementation was further hindered by hierarchical power dynamics, data silos caused by non-interoperable IT systems, and a lack of organisational readiness. Conversely, success was facilitated by authentic collaboration in policy design, proactive recruitment strategies valuing lived experience, and leadership that actively championed the role. Ultimately, the review indicates that without addressing the structural precarity of the workforce—specifically regarding pay and integration—CHW programmes remain vulnerable to high turnover and limited sustainability.

Given the scale of this review, a wide variety of divergent experiences was evident across findings relating to policy, certification, recruitment, IT and data systems, partnerships, and motivation, where similar conditions yielded converse outcomes. For instance, formal certification was found to legitimise the workforce in some contexts [88, 101], but functioned as a barrier to recruitment by excluding experienced grassroots candidates in others [78]. Similarly, digital infrastructure tools had a mixed impact: electronic health record access facilitated communication with providers [52], whereas a lack of database interoperability or restricted system access created double documentation burdens and forced a reliance on unintegrated spreadsheets [52, 116]. These contrasting experiences appear to be shaped by several intersecting factors, including the structural differences between statutory health frameworks and community settings, or the degree to which a deployment relies on top-down versus bottom-up design. Rather than indicating simple variation, this heterogeneity underscores the context-dependent nature of CHW programmes along with the variation in the manner that the workers are deployed.

### Comparison with previous research

The implementation determinants identified in this review demonstrate a notable alignment with the wider global literature on CHW deployment. Syntheses spanning varied economic and geographic contexts, such as those by Scott et al. [3], Kok et al. [49], and the World Health Organization [5], have consistently documented that CHW integration is heavily shaped by the interplay of overarching policy environments, institutional readiness, and frontline clinical relationships. The fact that contemporary findings from high-income settings mirror these older syntheses suggests that the core health service architectures governing lay health work have remained largely unchanged over time. A prominent example of this continuity is the misalignment between the CHW scope of practice and available funding mechanisms. While reviews such as Viswanathan et al. [123] established the efficacy of CHWs in increasing healthcare utilisation, the findings of this synthesis suggest that structural and financial barriers to sustainable deployment remain a primary hurdle. In HICs, the challenge appears to stem less from an absolute scarcity of funds and more from the bureaucratic structure of existing reimbursement models, which can be poorly aligned with preventative, community-based care [74]. This is further complicated by high heterogeneity in evaluation methods, which can make it difficult for policymakers to draw generalisable conclusions about value for money [124].

Our findings extend this by highlighting how product-focused metrics actively undermine workforce stability, forcing organisations to terminate contracts with effective CHWs simply because they miss narrow quantitative targets [59]. This operational tension is reinforced by contemporary survey data from primary care clinicians, which indicates that healthcare practices frequently restrict CHWs to transactional tasks such as discrete screenings rather than longitudinal care, an issue closely tied to limited billing uptake and technical integration hurdles [105]. This contrast with many LMIC contexts, where funding challenges often relate to the scarcity of any funds, underscores the distinct HIC challenge of navigating complex reimbursement models. While this funding dynamic is heavily shaped by the dominance of US studies within our sample, the underlying tension between transactional structures and relational care is highly relevant to other HIC settings. In the US, this barrier is driven primarily by multi-payer insurance billing requirements [74], whereas in nationalised systems like those of the UK or Australia, it typically stems from a reliance on non-recurrent public health or local government allocations. Despite these architectural differences, the frontline operational result across OECD settings is consistent: a cyclical pattern of short-term commissioning that breeds workforce precarity and high turnover.

This institutional precarity directly impacts individual employment conditions, with the review providing clear evidence linking low compensation to workforce attrition. Although intrinsic motivation remains a powerful driver among CHWs [54, 73, 81], this synthesis incorporates quantitative evidence demonstrating that dissatisfaction with pay and organisational support are among the strongest predictors of a CHW’s intent to leave the workforce [82]. Long-scale survey data thus provide a quantifiable operational risk, yet the small number of quantitative data streams across the wider review sample means these statistical trends must be interpreted alongside the rich contextual nuances provided by the qualitative findings. Data indicating that a significant proportion of CHWs earn salaries near the poverty line [108] suggest that the workforce is often economically aligned with the populations they serve. This finding aligns with the WHO’s global guideline advocating for fair remuneration [5]. It also suggests that HICs, despite their resources, face ongoing difficulties in professionalising the workforce effectively compared to some established LMIC national programmes, such as Brazil’s *Sistema Único de Saúde* or Unified Health System (SUS) [125].

Integration of CHWs into clinical teams remains an equally persistent challenge. Consistent with implementation science literature on organisational readiness [126, 127], this review found that leadership buy-in is essential, but top-down leadership is insufficient if it ignores hierarchical power dynamics on the frontline [69]. The unique position of CHWs—bridging the gap between a client and a clinical provider—creates inherent friction within traditional medical hierarchies [94, 115]. Effective integration therefore requires protocols that explicitly address and manage these power dynamics through proactive efforts to facilitate communication [69, 74], ensuring the CHW’s voice is heard and respected within the clinical team [70]. Importantly, while the macro-level funding architectures differ fundamentally between nationalised and multi-payer systems, frontline implementation barriers show a notable convergence. Clinical scepticism regarding lay roles and hierarchical power dynamics are features of modern Western medicine that transcend national healthcare boundaries. Whether a CHW is embedded within a US safety-net clinic or a UK Primary Care Network (PCN), they face a similar struggle to establish legitimacy alongside licensed clinical staff who are trained within traditionally professionalised, risk-averse structures. This is particularly relevant to the UK context, where PCNs and initiatives like the Community Health and Wellbeing Worker (CHWW) model, which emulates Brazil’s highly integrated and geographically-based CHW model, are attempting to standardise integration [19, 128]. These efforts signal an intention to shift towards household-centred care, yet they remain highly susceptible to the exact same professional hierarchies and boundary maintenance identified in the American literature.

These professional boundaries are frequently reinforced by the technical infrastructure of health services. For instance, this review identified several challenges associated with operationalising digital tools for complex CHW interventions. IT systems often acted as barriers due to a lack of interoperability, forcing CHWs to perform “double documentation” or navigate systems designed for billing rather than care coordination [52, 74]. These challenges are not unique to CHWs but reflect broader systemic digital barriers [129]. Our findings suggest that legacy administrative platforms can reduce relational capacity by prioritising rigid data entry over patient contact, reflecting a distinct HIC barrier where complex, established infrastructure is poorly configured for a lay workforce [52, 116].

#### Implications for practice and policy

Translating these findings into health service design would benefit from structural adjustments across financing and deployment frameworks. First, sustainable funding models should consider transitioning from transactional, short-term mechanisms to structures that better accommodate relational care. In multi-payer configurations, this might involve moving away from episodic fee-for-service models toward value-based payment architectures or capitated global budgets that explicitly account for outreach and care coordination [74]. Within single-payer frameworks like the UK’s NHS, sustainability could be enhanced by moving away from discretionary grants and integrating workforce funding into the core baseline allocations of Integrated Care Systems or long-term PCN contracts [19, 128]. Furthermore, to maintain workforce equity and diversity, evolving state credentialing and certification frameworks should ideally incorporate flexible, portfolio-based assessment pathways that recognise lived experience and community trust alongside formal academic qualifications [59].

Frontline implementation would also likely benefit from clearer operational protocols to manage clinical team and technical integration. Rather than dropping workers into existing practices without structural adjustment, primary care services could introduce formal multidisciplinary frameworks that clearly define complementary scopes of practice and look to mitigate traditional medical hierarchies [70]. This clinical integration might be further supported by technical infrastructure updates. Health service providers should consider involving CHWs directly in the design and customisation of electronic health record workflows, ensuring that systems are optimised to capture qualitative social determinants of health rather than narrow clinical data points [52, 116]. Finally, healthcare employers could address workforce attrition by establishing structural care frameworks, where reflective, peer-supported supervision is offered as a protected component of standard working hours to help alleviate burnout and vicarious trauma [107, 130].

### Future research directions

Future research should move toward evaluative studies of solutions, such as the impact of certification policies on workforce diversity and retention—testing the hypothesis that increased professionalisation might inadvertently exclude community-embedded candidates. As the cost-effectiveness of CHW interventions in certain HIC contexts remains under-researched [124], robust economic analyses are essential. Van Iseghem et al. [124] highlight that sustainable funding must account for the social embeddedness of the role, supporting the argument that future evaluations should adopt a societal perspective to capture the full breadth of CHW impact, including contributions to poverty reduction and community empowerment.

#### Strengths and limitations

A key strength of this review was the use of the Kok et al. [49] framework as a foundation, which grounded our analysis in established evidence of CHW performance while allowing for the inductive generation of new themes. Further strengths include the prospective registration of the study protocol, a comprehensive multi-database search strategy, and the use of independent dual screening and extraction to ensure procedural rigour. Furthermore, while previous reviews in this field have largely relied on narrative or scoping methodologies, a distinct strength of this study is the application of the GRADE-CERQual framework specifically to HIC implementation data. This allows us to move beyond thematic description and explicitly weight the empirical certainty of our findings for the benefit of health service commissioners.

Limitations include the absence of forward citation and grey literature searching, as well as evidence in languages other than English, due to resource constraints, meaning some programmes may have been missed. The broad scope of the review also increased the likelihood that some relevant studies were not captured. However, these omissions were mitigated by the substantial volume of included studies and the data saturation achieved, with the high GRADE-CERQual confidence ratings further indicating that the primary findings are robust and unlikely to be altered by additional research. Geographically, the evidence base was heavily dominated by studies from the US, whose specific insurance-based healthcare landscape may not be directly transferable to countries with nationalised systems like the UK or Canada. A final limitation is that while the review captured the perspectives of healthcare leaders and frontline CHWs, the voice of the service user remained under-represented.

## Conclusion

This review reveals a central paradox: while the CHW workforce is increasingly recognised as essential infrastructure—a status highlighted by the COVID-19 pandemic—it is simultaneously undermined by systemic fragility. Funding models, data systems, and professional hierarchies remain ill-equipped to support relational, community-centred work [High certainty]. Sustainability depends on a fundamental shift in health system design. Systems must adapt to protect and professionalise this cadre, adhering to the principles advocated by the Community Health Impact Coalition (CHIC) [130], ensuring CHWs are salaried, skilled, supervised, and supplied [Moderate certainty]. Moving toward sustainable financing and genuine integration is required to value trust-building as highly as clinical outputs [High certainty].

## Supplementary Information

Below is the link to the electronic supplementary material.

Supplementary Material 1: Additional File 1.docx: Containing ‘Additional Resource 1: Database Search Strategies’, ‘Additional Resource 2: Mixed Methods Appraisal Tool (MMAT) (2018) Quality Criteria’, and ‘Additional Resource 3: GRADE-CERQual assessment’.

Supplementary Material 2: Additional File 2.xlsx: Containing full and complete Microsoft Excel data extraction sheet.

## Data Availability

The datasets generated and/or analysed during the current study, including full search strings and data extraction tables, are included in supplementary information files.

## Abbreviations

BFFS: Best Fit Framework Synthesis
CBO: Community-Based Organisation
CHIC: Community Health Impact Coalition
CHW: Community Health Worker
CHWW: Community Health and Wellbeing Worker
EHR: Electronic Health Record
GP: General Practitioner
GRADE-CERQual: Grading of Recommendations Assessment, Development and Evaluation – Confidence in the Evidence from Reviews of Qualitative research
HIC: High-Income Country
IT: Information Technology
LLM: Large Language Model
LMIC: Low- and Middle-Income Country
MCO: Managed Care Organisation
MMAT: Mixed Methods Appraisal Tool
NHS: National Health Service
OECD: Organisation for Economic Co-operation and Development
PCN: Primary Care Network
PRISMA: Preferred Reporting Items for Systematic Reviews and Meta-Analyses
SUS: Sistema Único de Saúde / Unified Health System
UK: United Kingdom
US: United States

## References

[1] Zulu JM, Perry HB. Community health workers at the dawn of a new era. Health Res Policy Syst. 2021;19:1–5. 10.1186/s12961-021-00761-7 PMC8507089 34641904

[2] Perry HB, Zulliger R, Rogers MM. Community health workers in low-, middle-, and high-income countries: an overview of their history, recent evolution, and current effectiveness. Annu Rev Public Health. 2014;35(1):399–421. 10.1146/annurev-publhealth-032013-182354 24387091

[3] Scott K, Beckham SW, Gross M, Pariyo G, Rao KD, Cometto G, Perry HB. What do we know about community-based health worker programs? A systematic review of existing reviews on community health workers. Hum Resour Health. 2018;16:1–17. 10.1186/s12960-018-0304-x PMC6097220 30115074

[4] American Public Health Association. Support for community health workers to increase health access and to reduce health inequities. AHPA Policy No. 2009-1. 2009.

[5] World Health Organization. WHO guideline on health policy and system support to optimize community health worker programmes. Geneva: World Health Organization; 2018. 30431747

[6] Olaniran A, Smith H, Unkels R, Bar-Zeev S, van den Broek. N. Who is a community health worker?–a systematic review of definitions. Global Health Action. 2017;10(1):1272223. 10.1080/16549716.2017.1272223 PMC5328349 28222653

[7] Bhaumik S, Moola S, Tyagi J, Nambiar D, Kakoti M. Community health workers for pandemic response: a rapid evidence synthesis. BMJ Global Health. 2020;5(6):e002769. 10.1136/bmjgh-2020-002769 PMC7292038 32522738

[8] Gupta A, Khan S. Importance of Community Health Workers for Maternal Health Care Management. Public Health Rev. 2024;45:1606803. 10.3389/phrs.2024.1606803 PMC10917972 38455869

[9] Levitt NS, Puoane T, Denman CA, Abrahams-Gessel S, Surka S, Mendoza C, Khanam M, Alam S, Gaziano TA. Referral outcomes of individuals identified at high risk of cardiovascular disease by community health workers in Bangladesh, Guatemala, Mexico, and South Africa. Global Health Action. 2015;8(1):26318. 10.3402/gha.v8.26318 PMC4390559 25854780

[10] Lewin S, Munabi-Babigumira S, Glenton C, Daniels K, Bosch-Capblanch X, Van Wyk BE, Odgaard-Jensen J, Johansen M, Aja GN, Zwarenstein M. Lay health workers in primary and community health care for maternal and child health and the management of infectious diseases. Cochrane Database Syst Rev. 2010;(3). 10.1002/14651858.CD004015.pub3 PMC6485809 20238326

[11] Mishra SR, Neupane D, Preen D, Kallestrup P, Perry HB. Mitigation of non-communicable diseases in developing countries with community health workers. Globalization Health. 2015;11:1–5. 10.1186/s12992-015-0129-5 PMC4641398 26555199

[12] World Health Organization. What do we know about community health workers? A systematic review of existing reviews. Geneva: World Health Organization; 2020.

[13] World Health Organization. Health workforce. 2025. https://www.who.int/health-topics/health-workforce/. Accessed 10 October 2025.

[14] Knowles M, Crowley AP, Vasan A, Kangovi S. Community health worker integration with and effectiveness in health care and public health in the United States. Annu Rev Public Health. 2023;44(1):363–81. 10.1146/annurev-publhealth-071521-031648 37010928

[15] Lombardi BM, Lombardi B, Galloway E, Zerden LS. Understanding Available Data Sources to Estimate the Size and Distribution of Community Health Workers in the United States. Med Care Res Rev. 2025;82(3):276–83. 10.1177/10775587241304145 PMC12018715 40271967

[16] Torres S, Labonté R, Spitzer DL, Andrew C, Amaratunga C. Improving health equity: the promising role of community health workers in Canada. Healthc Policy. 2014;10(1):73. PMC4253897 25410697

[17] McDermott RA, Schmidt B, Preece C, Owens V, Taylor S, Li M, Esterman A. Community health workers improve diabetes care in remote Australian Indigenous communities: results of a pragmatic cluster randomized controlled trial. BMC Health Serv Res. 2015;15(1):68. 10.1186/s12913-015-0695-5 PMC4337108 25884300

[18] Masquillier C, Cosaert T. Facilitating access to primary care for people living in socio-economically vulnerable circumstances in Belgium through community health workers: towards a conceptual model. BMC Prim Care. 2023;24(1):281. 10.1186/s12875-023-02214-2 PMC10731868 38114909

[19] Junghans C, Antonacci G, Williams A, Harris M. Learning from the universal, proactive outreach of the Brazilian Community Health Worker model: impact of a Community Health and Wellbeing Worker initiative on vaccination, cancer screening and NHS health check uptake in a deprived community in the UK. BMC Health Serv Res. 2023;23(1):1092. 10.1186/s12913-023-10084-8 PMC10568890 37821938

[20] Javanparast S, Windle A, Freeman T, Baum F. Community health worker programs to improve healthcare access and equity: are they only relevant to low-and middle-income countries? Int J Health Policy Manage. 2018;7(10):943. 10.15171/ijhpm.2018.53 PMC6186464 30316247

[21] Najafizada SAM, Bourgeault IL, Labonte R, Packer C, Torres S. Community health workers in Canada and other high-income countries: A scoping review and research gaps. Can J Public Health. 2015;106:e157–64. 10.17269/CJPH.106.4747 PMC6972431 26125243

[22] Vanden Bossche D, Lagaert S, Willems S, Decat P. Community health workers as a strategy to tackle psychosocial suffering due to physical distancing: a randomized controlled trial. Int J Environ Res Public Health. 2021;18(6):3097. 10.3390/ijerph18063097 PMC8002631 33802870

[23] Kim K, Choi JS, Choi E, Nieman CL, Joo JH, Lin FR, Gitlin LN, Han H-R. Effects of community-based health worker interventions to improve chronic disease management and care among vulnerable populations: a systematic review. Am J Public Health. 2016;106(4):e3–28. 10.2105/AJPH.2015.302987 PMC4785041 26890177

[24] Amagyei A, Meal A, Shaw I, Adams G. Effectiveness of community health worker-led diabetes self-management education on type 2 diabetes patients: a systematic review and meta-analysis. Int J Diabetes. 2020;1(2):40–50.

[25] Bellhouse S, McWilliams L, Firth J, Yorke J, French DP. Are community-based health worker interventions an effective approach for early diagnosis of cancer? A systematic review and meta‐analysis. Psycho‐oncology. 2018;27(4):1089–99. 10.1002/pon.4575 29080378

[26] Wells KJ, Luque JS, Miladinovic B, Vargas N, Asvat Y, Roetzheim RG, Kumar A. Do community health worker interventions improve rates of screening mammography in the United States? A systematic review. Cancer Epidemiol Biomarkers Prev. 2011;20(8):1580–98. 10.1158/1055-9965.EPI-11-0276 PMC3153589 21653645

[27] Parekh TM, Copeland CR, Dransfield MT, Cherrington A. Application of the community health worker model in adult asthma and COPD in the US: a systematic review. BMC Pulm Med. 2019;19:1–8. 10.1186/s12890-019-0878-7 PMC6593583 31242944

[28] Han H-R, Kim K, Murphy J, Cudjoe J, Wilson P, Sharps P, Farley JE. Community health worker interventions to promote psychosocial outcomes among people living with HIV—A systematic review. PLoS ONE. 2018;13(4):e0194928. 10.1371/journal.pone.0194928 PMC5915269 29689054

[29] Carter-Pokras OD, Jaschek G, Martinez IL, Brown PB, Mora SE. Newton, N, and Luciani, I. Perspectives on Latino lay health promoter programs: Maryland, 2009. Am J Public Health. 2011;101(12):2281–6. 10.2105/AJPH.2011.300317 PMC3222417 22021305

[30] Henderson S, Kendall E. Community navigators’: making a difference by promoting health in culturally and linguistically diverse (CALD) communities in Logan, Queensland. Aust J Prim Health. 2011;17(4):347–54. 10.1071/PY11053 22112703

[31] Visram S. Impact and acceptability of lay health trainer-led lifestyle interventions delivered in primary care: a mixed method study. Prim Health Care Res Dev. 2017;18(4):333–43. 10.1017/S146342361700010X 28393741

[32] Assefa Y, Gelaw YA, Hill PS, Taye BW, Van Damme. W. Community health extension program of Ethiopia, 2003–2018: successes and challenges toward universal coverage for primary healthcare services. Globalization Health. 2019;15(1):24. 10.1186/s12992-019-0470-1 PMC6434624 30914055

[33] Castro MC, Massuda A, Almeida G, Menezes-Filho NA, Andrade MV, de Souza Noronha KVM, Rocha R, Macinko J, Hone T, Tasca R. Brazil’s unified health system: the first 30 years and prospects for the future. Lancet. 2019;394(10195):345–56. 10.1016/S0140-6736(19)31243-7 31303318

[34] Ahmed S, Chase LE, Wagnild J, Akhter N, Sturridge S, Clarke A, Chowdhary P, Mukami D, Kasim A, Hampshire K. Community health workers and health equity in low-and middle-income countries: systematic review and recommendations for policy and practice. Int J Equity Health. 2022;21(1):49. 10.1186/s12939-021-01615-y PMC8996551 35410258

[35] Glenton C, Javadi D, Perry HB. Community health workers at the dawn of a new era: 5. Roles and tasks. Health Res Policy Syst. 2021;19:1–16. 10.1186/s12961-021-00748-4 PMC8506082 34641903

[36] McElfish PA, Sorrell S, Marin LP, Pile J, Huff A, Faitak B, Moore S, Ayala A, Bonilla S, Vargas CN. Integrating Community Health Workers Into Clin Settings Health Expect. 2026;29(1):e70604. 10.1111/hex.70604 PMC12914219 41704156

[37] Rowe JT, Parrillo E, Stanford O, Wenzel J, Johnston FM. Individual and systemic barriers blocking community health workers from helping the seriously Ill. J Palliat Med. 2024;27(3):358–66. 10.1089/jpm.2022.0516 PMC10903179 38010809

[38] Hodgins S, Kok M, Musoke D, Lewin S, Crigler L, LeBan K, Perry HB. Community health workers at the dawn of a new era: 1. Introduction: tensions confronting large-scale CHW programmes. Health Res Policy Syst. 2021;19:1–21. 10.1186/s12961-021-00752-8 PMC8506102 34641886

[39] Ballard M, Johnson A, Mwanza I, Ngwira H, Schechter J, Odera M, Mbewe DN, Moenga R, Muyingo P, Jalloh R. Community health workers in pandemics: evidence and investment implications. Glob Health Sci Pract. 2022;10(2). 10.9745/GHSP-D-21-00648 PMC9053152 35487542

[40] Hanson K, Brikci N, Erlangga D, Alebachew A, De Allegri M, Balabanova D, Blecher M, Cashin C, Esperato A, Hipgrave D. The Lancet Global Health Commission on financing primary health care: putting people at the centre. Lancet Global Health. 2022;10(5):e715–72. 10.1016/S2214-109X(22)00005-5 PMC9005653 35390342

[41] Page MJ, McKenzie JE, Bossuyt PM, Boutron I, Hoffmann TC, Mulrow CD, Shamseer L, Tetzlaff JM, Akl EA, Brennan SE, et al. The PRISMA 2020 statement: an updated guideline for reporting systematic reviews. BMJ. 2021;372. 10.1136/bmj.n71 PMC8005924 33782057

[42] OECD. Members and partners. 2024. https://www.oecd.org/en/about/members-partners.html. Accessed 10 October 2025.

[43] World Health Organization. The World Health Report 2001: Mental health: new understanding, new hope. Geneva: World Health Organization; 2001.

[44] World Health Organization. The World Health Report 2006: Working together for health. Geneva: World Health Organization; 2006.

[45] Ouzzani M, Hammady H, Fedorowicz Z, Elmagarmid A. Rayyan—a web and mobile app for systematic reviews. Syst Reviews. 2016;5(1):210. 10.1186/s13643-016-0384-4 PMC5139140 27919275

[46] Hong QN, Fàbregues S, Bartlett G, Boardman F, Cargo M, Dagenais P, Gagnon M-P, Griffiths F, Nicolau B, O’Cathain A. The Mixed Methods Appraisal Tool (MMAT) version 2018 for information professionals and researchers. Educ Inform. 2018;34(4):285–91.

[47] Booth A, Carroll C. How to build up the actionable knowledge base: the role of ‘best fit’framework synthesis for studies of improvement in healthcare. BMJ Qual Saf. 2015;24(11):700–8. 10.1136/bmjqs-2014-003642 PMC4680127 26306609

[48] Carroll C, Booth A, Leaviss J, Rick J. Best fit framework synthesis: refining the method. BMC Med Res Methodol. 2013;13:1–16. 10.1186/1471-2288-13-37 PMC3618126 23497061

[49] Kok MC, Dieleman M, Taegtmeyer M, Broerse JE, Kane SS, Ormel H, Tijm MM, De Koning KA. Which intervention design factors influence performance of community health workers in low-and middle-income countries? A systematic review. Health Policy Plann. 2015;30(9):1207–27. 10.1093/heapol/czu126 PMC4597042 25500559

[50] Stern C, Lizarondo L, Carrier J, Godfrey C, Rieger K, Salmond S, Apóstolo J, Kirkpatrick P, Loveday H. Methodological guidance for the conduct of mixed methods systematic reviews. JBI Evid Synthesis. 2020;18(10):2108–18. 10.11124/JBISRIR-D-19-00169 32813460

[51] Noyes J, Booth A, Moore G, Flemming K, Tunçalp Ö, Shakibazadeh E. Synthesising quantitative and qualitative evidence to inform guidelines on complex interventions: clarifying the purposes, designs and outlining some methods. BMJ Global Health. 2019;4(Suppl 1):pe000893. 10.1136/bmjgh-2018-000893 PMC6350750 30775016

[52] Acharya H, Sykes KJ, Neira TM, Scott A, Pacheco CM, Sanner M, Ablah E, Oyowe K, Ellerbeck EF, Greiner KA. A novel electronic record system for documentation and efficient workflow for community health workers: development and usability study. JMIR Formative Res. 2024;8(1):e52920. 10.2196/52920 PMC11019415 38557671

[53] Adinkrah EK, Bazargan S, Cobb S, Kibe LW, Vargas R, Waller J, Sanchez H, Bazargan M. Mobilizing faith-based COVID-19 health ambassadors to address COVID-19 health disparities among African American older adults in under-resourced communities: A hybrid, community-based participatory intervention. PLoS ONE. 2024;19(2):e0285963. 10.1371/journal.pone.0285963 PMC10868749 38358995

[54] Agomo CN. The narratives of natural helpers: a qualitative exploration of the lived experiences of community health workers in Alabama [dissertation]. Tuscaloosa (AL): The University of Alabama; 2024.

[55] Allen CG, Escoffery C, Satsangi A, Brownstein JN. Strategies to improve the integration of community health workers into health care teams: a little fish in a big pond. Prev Chronic Dis. 2015;12. 10.5888/pcd12.150199 PMC4576500 26378900

[56] Apers H, Masquillier C. Facilitators and Barriers in Collaborations Between Community Health Workers with Primary and Well-Being Providers in Primary Healthcare in Belgium. Healthcare. 2024;12(23):p2348. 10.3390/healthcare12232348 PMC11641491 39684970

[57] AuYoung M, Rodriguez Espinosa P, Chen WT, Juturu P, Young ME, Casillas A, Adkins-Jackson P, Hopfer S, Kissam E, Alo AK, et al. Addressing racial/ethnic inequities in vaccine hesitancy and uptake: lessons learned from the California Alliance Against COVID-19. J Behav Med. 2023;46(1):153–66. 10.1007/s10865-022-00284-8 PMC8783654 35066696

[58] Ban A, Shrestha A, Van den Berk-Clark C, Ballard J, Logan R Jr, Logan Sr T, Francioni A, Murray M, Baker EA. Through the eyes of community health workers: what was needed to increase COVID-19 vaccine uptake in the Missouri Southeast region. Front Public Health. 2024;12:1286177. 10.3389/fpubh.2024.1286177 PMC11004485 38601509

[59] Billimek J, Michelen M, Cantero PJ, Chirinos N, Salazar R, Foo MA, Peralta S, Morey BN, Chow JJ, Kauffman V, et al. People versus product: conditions for success for community health workers as sustainable members of the public health workforce. Milbank Q. 2025. 10.1111/1468-0009.70038 PMC12864003 40716100

[60] Brandford A, Sanchez M, Pitones P, Bolin J. From the ground up: building and implementing a successful CHW/promotor(a) program for cancer screening, training, education, and prevention. Health Promot Pract. 2025:15248399241308198. 10.1177/15248399241308198 39760400

[61] Bridges KM, LeMaster JW, Parente DJ, Pacheco CM, Schultz C, Morrow E, Corriveau E, Miras Neira T, Greiner KA, Woodward J. Assessing Social Needs and Engaging Community Health Workers in Underserved Kansas Counties: Insights From Primary Care Providers and Clinic Managers. J Prim Care Community Health. 2023;14:21501319231214513. 10.1177/21501319231214513 PMC10693805 38041409

[62] Carson SL, Hong C, Behforouz H, Chang E, Dixon LZ, Factor D, George SM, Lewis J, Majeno A, Morales M. Mechanisms for Community Health Worker action on patient-, institutional-, and community-level barriers to primary care in a safety-net setting. J Ambul Care Manag. 2022;45(1):22–35. 10.1097/JAC.0000000000000405 PMC8622376 34812754

[63] Casillas A, Rosas LG, Carson SL, Orechwa A, North G, AuYoung M, Kim G, Guereca JA, Ramers CB, Burke NJ. STOP COVID-19 CA: community engagement to address the disparate impacts of the COVID-19 pandemic in California. Front Health Serv. 2022;2:935297. 10.3389/frhs.2022.935297 PMC10012632 36925779

[64] Conway J, Tsourtos G, Lawn S. The barriers and facilitators that indigenous health workers experience in their workplace and communities in providing self-management support: a multiple case study. BMC Health Serv Res. 2017;17:1–13. 10.1186/s12913-017-2265-5 PMC5415721 28468612

[65] Cook T, Wills J. Engaging with marginalized communities: the experiences of London health trainers. Perspect Public health. 2012;132(5):221–7. 10.1177/1757913910393864 22991369

[66] Coritsidis A, Arniella G, Berhane T, Madden D, Ruiz J, Vangeepuram N, Oliver K. Sustaining Communities and Community Health Workers: Lessons Learned From Community Health Workers’ Experience During the COVID-19 Pandemic. J Ambul Care Manag. 2025;48(3):159–68. 10.1097/JAC.0000000000000532 40397814

[67] D’Arcy C, Taket A, Hanna L. Implementing empowerment-based Lay Health Worker programs: a preliminary study. Health Promot Int. 2019;34(4):726–34. 10.1093/heapro/day023 29688341

[68] Foo CY, Potter K, Nielsen L, Rohila A, Maravic MC, Schnitzer K, Pachas GN, Levy DE, Reyering S, Thorndike AN. Implementation of community health worker support for tobacco cessation: a mixed-methods study. Psychiatric Serv. 2025;76(1):30–40. 10.1176/appi.ps.20240044 PMC13231508 39118574

[69] Fuller SM, Arnold EA, Xavier J, Ibe CA, Steward WT, Myers JJ, Rebchook G, Koester KA. Integrating community health workers into HIV care clinics: a qualitative study with health system leaders and clinicians in the Southern United States. BMC Health Serv Res. 2024;24(1):1339. 10.1186/s12913-024-11381-6 PMC11531124 39487486

[70] George PE, Haughton J, Whiskey R, Levano S, Muleta H, Fiori KP. Barriers and facilitators to the implementation of a health-related social needs screening and referral intervention in the Bronx. J Prim Care Community Health. 2025;16:21501319251334210. 10.1177/21501319251334210 PMC12126658 40448351

[71] George R, Gunn R, Wiggins N, Rowland R, Davis MM, Maes K, Kuzma A, McConnell KJ. Early lessons and strategies from statewide efforts to integrate community health workers into Medicaid. J Health Care Poor Underserved. 2020;31(2):845. 10.1353/hpu.2020.0064 PMC7791094 33410811

[72] Gore R, Brown A, Wong G, Sherman S, Schwartz M, Islam N. Integrating community health workers into safety-net primary care for diabetes prevention: qualitative analysis of clinicians’ perspectives. J Gen Intern Med. 2020;35:1199–210. 10.1007/s11606-019-05581-8 PMC7174477 31848857

[73] Grice-Jackson T, Rogers I, Ford E, Dickinson R, Frere-Smith K, Goddard K, Silver L, Topham C, Nahar P, Musinguzi G. A community health worker led approach to cardiovascular disease prevention in the UK—SPICES-Sussex (scaling-up packages of interventions for cardiovascular disease prevention in selected sites in Europe and Sub-saharan Africa): an implementation research project. Front Health Serv. 2024;4:1152410. 10.3389/frhs.2024.1152410 PMC11113076 38784704

[74] Gunter KE, Ellingson MK, Nieto M, Jankowski R, Tanumihardjo JP. Barriers and strategies to operationalize Medicaid reimbursement for CHW services in the state of Minnesota: a case study. J Gen Intern Med. 2023;38(Suppl 1):70–7. 10.1007/s11606-022-07925-3 PMC10043123 36864272

[75] Haughton J, Takemoto ML, Schneider J, Hooker SP, Rabin B, Brownson RC, Arredondo EM. Identifying barriers, facilitators, and implementation strategies for a faith-based physical activity program. Implement Sci Commun. 2020;1(1):51. 10.1186/s43058-020-00043-3 PMC7427873 32885207

[76] Hernandez-Salinas C, Marsiglia FF, Oh H, Campos AP, Rosa DL. Community health workers as puentes/bridges to increase COVID-19 health equity in Latinx communities of the southwest US. J Community Health. 2023;48(3):398–413. 10.1007/s10900-022-01182-5 PMC9762869 36536085

[77] Hood QO, Irvine N, Shah K, Ali SH, Mezzacca TA, Serrano M, Thorpe LE, Huang TT, Khan MR, Islam N. A qualitative exploration of the implementation facilitators and challenges of a community health worker program to address health disparities for people recently released from incarceration using the consolidated framework for implementation research. Implement Sci Commun. 2024;5(1):118. 10.1186/s43058-024-00653-1 PMC11490189 39425238

[78] Ingram M, Medrano A, Encinas D, Itule-Klasen L, Coronado G, Esparza M, Cordova RV, Seline J, Addington RC, Wilkinson-Lee AM. A Case Study of CHW Integration in Two Health Departments in Southern Arizona. J Ambul Care Manag. 2025;48(3):147–58. 10.1097/JAC.0000000000000529 40345141

[79] Johnson CM, Allicock MA, Sharkey JR, Umstattd Meyer MR, Gómez L, Prochnow T, Laviolette C, Beltrán E, Garza LM. Promotoras de salud in a father-focused nutrition and physical activity program for border communities: Approaches and lessons learned from collaboration. Int J Environ Res Public Health. 2022;19(18):11660. 10.3390/ijerph191811660 PMC9517298 36141933

[80] King C, Goldman A, Gampa V, Smith C, Muskett O, Brown C, Malone J, Sehn H, Curley C, Begay M-G. Strengthening the role of community health representatives in the Navajo Nation. BMC Public Health. 2017;17(1):348. 10.1186/s12889-017-4263-2 PMC5399395 28431541

[81] Kirimi DM. Assessing community health workers' performance motivation [dissertation]. Kent (OH): Kent State University; 2024.

[82] Kirkland C, Dill JS, Karnik H. Retention of community health workers in the public health workforce: public health workforce interests and needs survey, 2017 and 2021. Am J Public Health. 2024;114(1):44–7. 10.2105/AJPH.2023.307462 PMC10726946 38033282

[83] Koskan A, Friedman DB, Messias DK, Brandt HM, Walsemann K. Sustainability of promotora initiatives: program planners’ perspectives. J Public Health Manage Pract. 2013;19(5):E1–9. 10.1097/PHH.0b013e318280012a PMC3827959 23295409

[84] Kuroda M, Shaw AV, Campagna CD. The experiences of community health workers when communicating with refugees about COVID-19 vaccines in Syracuse, NY: a qualitative study. Heliyon. 2024;10(4). 10.1016/j.heliyon.2024.e26136 PMC10906162 38434037

[85] Lapidos A, Kieffer EC, Guzmán R, Hess K, Flanders T, Heisler M. Barriers and facilitators to community health worker outreach and engagement in Detroit, Michigan: a qualitative study. Health Promot Pract. 2022;23(6):1094–104. 10.1177/15248399211031818 34549635

[86] Mason T, Wilkinson GW, Nannini A, Martin CM, Fox DJ, Hirsch G. Winning policy change to promote community health workers: lessons from Massachusetts in the health reform era. Am J Public Health. 2011;101(12):2211–6. 10.2105/AJPH.2011.300402 PMC3222440 22021281

[87] Mathers J, Taylor R, Parry J. The challenge of implementing peer-led interventions in a professionalized health service: a case study of the national health trainers service in England. Milbank Q. 2014;92(4):725–53. 10.1111/1468-0009.12090 PMC4266174 25492602

[88] McAlearney AS, Menser T, Sieck CJ, Sova LN, Huerta TR. Opportunities for community health worker training to improve access to health care for Medicaid enrollees. Popul Health Manage. 2020;23(1):38–46. 10.1089/pop.2018.0117 31140931

[89] McCarville E, Martin MA, Pratap P, Pinsker E, Seweryn SM, Peters KE. Understanding critical factors associated with integration of community health workers into health and hospital systems. J Interprof Care. 2024;38(3):507–16. 10.1080/13561820.2023.2183183 36946323

[90] McCutcheon J, Cheng I, Quinones S, Mahabaleshwarkar R, Denizard-Thompson N, Wiseman K, Taylor Y, Thomas SW, Palakshappa D. A Mixed-Methods Study to Understand Community Health Worker Integration With Health Care Teams. J Ambul Care Manag. 2025;48(1):25–38. 10.1097/JAC.0000000000000511 39110535

[91] Menser T, Swoboda C, Sieck C, Hefner J, Huerta T, McAlearney AS. A community health worker home visit program: Facilitators and barriers of program implementation. J Health Care Poor Underserved. 2020;31(1):370–81. 10.1353/hpu.2020.0028 32037337

[92] Morey BN, Michelen M, Phan M, Cardenas S, Foo MA, Cantero PJ, Peralta S, Chirinos N, Salazar R, Montiel GI. Structural supports and challenges for community health worker models: lessons from the COVID-19 response in orange County, California. SSM-Qualitative Res Health. 2025;7:100510. 10.1016/j.ssmqr.2024.100510 PMC12021443 40291451

[93] Obasanjo I, Scott A, Griffin M, Agyemang-Duah A, Westhoff C, Toney S, Shelton P. Understanding the public health role, motivations, and perceptions of community health workers deployed to low-income housing in Richmond, Virginia. Community Health Equity Res Policy. 2024;44(2):219–27. 10.1177/2752535X231154051 36716267

[94] Orpinas P, Matthew RA, Alvarez-Hernandez LR, Calva A, Bermúdez JM. Promotoras voice their challenges in fulfilling their role as community health workers. Health Promot Pract. 2021;22(4):502–11. 10.1177/1524839920921189 32419515

[95] Palmer-Wackerly AL, Chaidez V, Wayment C, Baker J, Adams A, Wheeler LA. Listening to the voices of community health workers: A multilevel, culture-centered approach to overcoming structural barriers in US Latinx communities. Qual Health Res. 2020;30(3):423–36. 10.1177/1049732319855963 31274052

[96] Paulson CA, Durazo EM, Purry LD, Covington AE, Bob BA, Peters RA, Torchia S, Beard B, McDermott LE, Lerner A. Adding a seat at the table: a case study of the provider's perspective on integrating community health workers at provider practices in California. Front Public Health. 2021;9. 10.3389/fpubh.2021.690067 PMC8581190 34778164

[97] Payne J, Razi S, Emery K, Quattrone W, Tardif-Douglin M. Integrating community health workers (CHWs) into health care organizations. J Community Health. 2017;42(5):983–90. 10.1007/s10900-017-0345-4 28391593

[98] Perez M, Ceballos V, Rubio N, Garcia J, Rubi D, Cabrera A, Milligan K, Kelley A. Defining case management and outreach: perspectives from community health workers in New Mexico. Cogent Social Sci. 2024;10(1):2306921.

[99] Polletta VL, LeBrón AM, Sifuentes MR, Mitchell-Bennett LA, Ayala C, Reininger BM. Facilitators and barriers of a chronic care management intervention addressing diabetes among Mexican-origin adults. Health Educ Behav. 2021;48(6):831–41. 10.1177/10901981211014431 34027711

[100] Porteny T, Alegría M, Del Cueto, Fuentes L, Markle SL, NeMoyer A, Perez GK. Barriers and strategies for implementing community-based interventions with minority elders: positive minds-strong bodies. Implement Sci Commun. 2020;1(1):1–13. 10.1186/s43058-020-00034-4 PMC7427860 32885198

[101] Rahman R, Ross AM, Chesna S, Rama Tatikola M, Hopwood S. Studying Executive Directors and Supervisors Views of Organizational and Policy-Level Challenges Faced by Community Health Workers. J Ambul Care Manage. 2021;44(4):250–63. 10.1097/JAC.0000000000000389 34120125

[102] Rodriguez Espinosa P, Vázquez E, AuYoung M, Zaldivar F, Cheney AM, Sorkin D, Zender R, Corchado CG, Burke NJ. Partnering with community health workers to address COVID-19 health inequities: experiences of the California Alliance Against COVID-19. Am J Public Health. 2024;114(1):S45–9. 10.2105/AJPH.2023.307471 PMC10785166 38207262

[103] Rodriguez NM, Ruiz Y, Meredith AH, Kimiecik C, Adeoye-Olatunde OA, Kimera LF, Gonzalvo JD. Indiana community health workers: challenges and opportunities for workforce development. BMC Health Serv Res. 2022;22(1):117. 10.1186/s12913-022-07469-6 PMC8792135 35086545

[104] Rogers EA, Manser ST, Cleary J, Joseph AM, Harwood EM, Call KT. Integrating community health workers into medical homes. Annals Family Med. 2018;16(1):14–20. 10.1370/afm.2171 PMC5758315 29311170

[105] Rohan EA, Townsend JS, Bermudez AT, Thompson HL, Holman DM, Reza A, Tharpe FS, Wennerstrom A. Engaging Community Health Workers in Primary Care Practices: Provider Understanding of Roles, Benefits, and Barriers. J Ambul Care Manag. 2024;47(3):154–67. 10.1097/JAC.0000000000000501 PMC12044569 38775653

[106] Rojas E, Gerber BS, Tilton J, Rapacki L, Sharp LK. Pharmacists’ perspectives on collaborating with community health workers in diabetes care. J Am Pharmacists Association. 2015;55(4):429–33. 10.1331/JAPhA.2015.14123 PMC5549615 26161485

[107] Sainz M, Lemon E, Flores YA, Woods-Jaeger B. Nos apoyamos mutuamente: Strategies to supporting community health workers addressing the emergency and chronic needs of Latinx families. Psychol Serv. 2024;21(3):626. 10.1037/ser0000842 38451705

[108] Scanlon M, Thomas MR, Tang Q, Gonzalvo JD, Ruiz Y, Hart M, Jackson J, Litzelman DK. A cross-sectional survey of community health workers and their roles in Indiana. BMC Health Serv Res. 2025;25(1):1037. 10.1186/s12913-025-13182-x PMC12329865 40775706

[109] Schriger SH, Knowles M, Daglieri T, Kangovi S, Beidas RS. Barriers and facilitators to implementing an evidence-based community health worker model. JAMA Health Forum. 2024. 10.1001/jamahealthforum.2024.0034 PMC10924240 38457130

[110] Sharma SV, McPherson HH, John J, Guerrero R, Tennyson SB, Banerjee D, Siddiqui N, Savannah S, Durand P, Gao A. Framework, design, and baseline data of a collective impact initiative to strengthen the Community Health Worker workforce ecosystem in Texas. BMC Public Health. 2025;25(1):630. 10.1186/s12889-025-21812-6 PMC11829481 39955497

[111] Sherman M, Covert H, Fox L, Lichtveld M. Successes and lessons learned from implementing community health worker programs in community-based and clinical settings: insights from the Gulf Coast. J Public Health Manage Pract. 2017;23:S85–93. 10.1097/PHH.0000000000000653 28961658

[112] Smithwick J, Nance J, Covington-Kolb S, Rodriguez A, Young M. Community health workers bring value and deserve to be valued too: Key considerations in improving CHW career advancement opportunities. Front Public Health. 2023;11:1036481. 10.3389/fpubh.2023.1036481 PMC10030954 36969656

[113] Stupplebeen DA, Sentell TL, Pirkle CM, Juan B, Barnett-Sherrill AT, Humphry JW, Yoshimura SR, Kiernan J, Hartz CP, Keliikoa LB. Community health workers in action: community-clinical linkages for diabetes prevention and hypertension management at 3 community health centers. Hawai’i J Med Public Health. 2019;78(6 Suppl 1):15. PMC6603891 31285963

[114] Sursely AM, Kazmerzak D, Appelgate W, Walkner LM, Hiros S, Hileman R, Haines H, Afifi RA. I feel like my job is to give patients hope-perspectives of Community Health Workers and employers in Iowa: a mixed methods study. BMC Health Serv Res. 2025;25(1):386. 10.1186/s12913-025-12536-9 PMC11910000 40089724

[115] Toney AM, Martin T, Sanchez S, Kelley MS, Palmer-Wackerly AL, Chaidez V. Examining the Macrosystem Level of Influence on Community Health Worker Effectiveness in the State of Nebraska: A Qualitative Approach. J Community Health. 2022;47(3):510–8. 10.1007/s10900-022-01075-7 PMC8894838 35244819

[116] Tredinnick-Rowe J, Byng R, Brown T, Chapman D. Piloting a community health and well-being worker model in Cornwall: a guide for implementation and spread. BMC Prim Care. 2024;25(1):367. 10.1186/s12875-024-02595-y PMC11476637 39407146

[117] Trejo G, Arcury TA, Grzywacz JG, Tapia J, Quandt SA. Barriers and facilitators for promotoras’ success in delivering pesticide safety education to Latino farmworker families: La Familia Sana. J Agromed. 2013;18(2):75–86. 10.1080/1059924X.2013.766143 23540298

[118] Van der Wees PJ, Friedberg MW, Guzman EA, Ayanian JZ, Rodriguez HP. Comparing the implementation of team approaches for improving diabetes care in community health centers. BMC Health Serv Res. 2014;14(1):1–11. 10.1186/s12913-014-0608-z PMC4264557 25468448

[119] Washburn DJ, Callaghan T, Schmit C, Thompson E, Martinez D, Lafleur M. Community health worker roles and their evolving interprofessional relationships in the United States. J Interprof Care. 2021:1–7. 10.1080/13561820.2021.1974362 34652982

[120] Wennerstrom A, Sugarman M, Haywood CG, Jindal D, True G. Roles and Responsibilities of Community Health Workers in Louisiana Medicaid Managed Care Organizations. J Ambul Care Manag. 2024;47(1):22–32. 10.1097/JAC.0000000000000483 37994511

[121] Wilkinson GW, Mason T, Hirsch G, Calista JL, Holt L, Toledo J, Zotter J. Community health worker integration in health care, public health, and policy: a partnership model. J Ambul Care Manag. 2016;39(1):2–11. 10.1097/JAC.0000000000000124 26650741

[122] Williamson MF, Song HJ, Dougherty L, Parcsi L, Barr ML. Qualitative case study: a pilot program to improve the integration of care in a vulnerable inner-city community. Int J Integr Care. 2022;22(2). 10.5334/ijic.6184 PMC9122010 35634255

[123] Viswanathan M, Kraschnewski JL, Nishikawa B, Morgan LC, Honeycutt AA, Thieda P, Lohr KN, Jonas DE. Outcomes and costs of community health worker interventions: a systematic review. Med Care. 2010;48(9):792–808. 10.1097/MLR.0b013e3181e35b51 20706166

[124] Van Iseghem T, Jacobs I, Vanden Bossche D, Delobelle P, Willems S, Masquillier C, Decat P. The role of community health workers in primary healthcare in the WHO-EU region: a scoping review. Int J Equity Health. 2023;22(1):134. 10.1186/s12939-023-01944-0 PMC10357780 37474937

[125] Krieger MGM, Wenham C, Nacif Pimenta D, Nkya TE, Schall B, Nunes AC, De Menezes A, Lotta G. How do community health workers institutionalise: An analysis of Brazil’s CHW programme. Glob Public Health. 2022;17(8):1507–24. 10.1080/17441692.2021.1940236 34161201

[126] Kirk MA, Kelley C, Yankey N, Birken SA, Abadie B, Damschroder L. A systematic review of the use of the Consolidated Framework for Implementation Research. Implement Sci. 2015;11:1–13. 10.1186/s13012-016-0437-z PMC4869309 27189233

[127] Meza RD, Triplett NS, Woodard GS, Martin P, Khairuzzaman AN, Jamora G, Dorsey S. The relationship between first-level leadership and inner-context and implementation outcomes in behavioral health: a scoping review. Implement Sci. 2021;16:1–21. 10.1186/s13012-021-01104-4 PMC8259113 34229706

[128] National Association of Primary Care. Community Health and Wellbeing Workers (CHWWs). 2026. https://napc.co.uk/chww/. Accessed 10 October 2025.

[129] Alotaibi N, Wilson CB, Traynor M. Enhancing digital readiness and capability in healthcare: a systematic review of interventions, barriers, and facilitators. BMC Health Serv Res. 2025;25(1):500. 10.1186/s12913-025-12663-3 PMC11969766 40186200

[130] Community Health Impact Coalition. About CHIC. 2025. https://joinchic.org/about/. Accessed 10 October 2025.

