# Supplementary material for: Barriers to and facilitators of the implementation of Community Health Worker programmes in high-income countries: a systematic review
Source: BMC Health Serv Res. 2026 Jul 17;26:1394. doi: 10.1186/s12913-026-15114-9 (PMC13660263; doi:10.1186/s12913-026-15114-9)
Supplement: Supplementary file 1 [file 12913_2026_15114_MOESM1_ESM.docx]

**Barriers to and facilitators of the implementation of** **Community Health Worker programmes in high-income countries: a systematic review**

Andrew Riley, Hannah Doughty, James Edward Hill, Clarissa Giebel, Matthew Harris, Nefyn H Williams

**Corresponding Author:**

Dr Andrew Riley, BA (Hons), MA, MSc, PhD

Department of Primary Care and Mental Health

Institute of Population Health

University of Liverpool

L69 3GL

United Kingdom

a.r.riley@liverpool.ac.uk

**Additional Resource 1: Database Search Strategies**

**Medline @ OVID**

CINHAL Plus

Scopus

APA PSYCHINFO

**Additional Resource 2: Mixed Methods Appraisal Tool (MMAT) (2018) Quality Criteria**

All questions answered ‘yes’, ‘no’, or can’t tell.

Screening questions for all study types:

- S1. Are there clear research questions?
- S2. Do the collected data allow to address the research questions?

Qualitative Appraisal Questions

- 1.1. Is the qualitative approach appropriate to answer the research question?
- 1.2. Are the qualitative data collection methods adequate to address the research question?
- 1.3. Are the findings adequately derived from the data?
- 1.4. Is the interpretation of results sufficiently substantiated by data?
- 1.5. Is there coherence between qualitative data sources, collection, analysis and interpretation?

Quantitative Appraisal Questions

- 4.1. Is the sampling strategy relevant to address the research question?
- 4.2. Is the sample representative of the target population?
- 4.3. Are the measurements appropriate?
- 4.4. Is the risk of nonresponse bias low?
- 4.5. Is the statistical analysis appropriate to answer the research question?

Mixed methods Appraisal Questions

- 5.1. Is there an adequate rationale for using a mixed methods design to address the research question?
- 5.2. Are the different components of the study effectively integrated to answer the research question?
- 5.3. Are the outputs of the integration of qualitative and quantitative components adequately interpreted?
- 5.4. Are divergences and inconsistencies between quantitative and qualitative results adequately addressed?
- 5.5. Do the different components of the study adhere to the quality criteria of each tradition of the methods involved?

**MMAT Appraisal**

**Qualitative studies**

| **Study** | **S1** | **S2** | **1.1** | **1.2** | **1.3** | **1.4** | **1.5** |
| --- | --- | --- | --- | --- | --- | --- | --- |
| Agomo (2024) | Yes | Yes | Yes | Yes (Two-round semi-structured interviews provided sufficient data depth) | Yes | Yes (Findings were substantiated by extensive participant quotes) | Yes (Strong coherence between the interpretive framework and the results) |
| Apers & Masquillier (2024) | Yes | Yes | Yes | Yes | Yes | Yes (Interpretations are supported by diverse stakeholder perspectives) | Yes (Clear coherence between the data collection and resulting thematic framework) |
| AuYoung et al. (2023) | Yes | Yes | Yes (Qualitative approach appropriate for documenting community-led strategies) | Yes (Multiple sources: town halls, focus groups, and phone banking) | Yes (Key lessons were logically derived from multi-site qualitative findings) | Yes (Recommendations are substantiated by specific community outreach examples) | Yes (Clear coherence between the community-engaged framework and results) |
| Ban et al. (2024) | Yes | Yes | Yes (Narrative analysis is suitable for assessing professional and personal experiences) | Yes | Yes | Yes (Results are well-substantiated by verbatim participant examples) | Yes (Coherence maintained between the grassroots context and emergent themes) |
| Billimek et al. (2025) | Yes | Yes | Yes | Yes (Large sample size of 60 CHWs across interviews and focus groups) | Yes | Yes (Interpretations are well-supported by thematic analysis) | Yes (Strong coherence between the CBPR methodology and thematic findings) |
| Bridges et al. (2023) | Yes | Yes | Yes | Yes (Interviewed a representative pool of providers and managers across 26 clinics) | Yes | Yes (Findings are supported by representative quotes from respondents) | Yes (Standard COREQ checklist used; strong coherence between methods and findings) |
| Carson et al. (2022) | Yes | Yes | Yes | Yes (8 focus groups with 42 participants ensured adequate data saturation) | Yes (Patterns across patient and CHW groups were logically analysed) | Yes (Themes are substantiated by comparative quotes from both groups) | Yes (Clear coherence between the safety-net context and role interpretations) |
| Carter-Pokras et al. (2011) | Yes | Yes | Yes | Yes | Yes | Yes (Findings were substantiated by direct observations from the 10 programs) | Yes (Strong coherence between bilingual data collection and NVivo analysis) |
| **Study** | **S1** | **S2** | **1.1** | **1.2** | **1.3** | **1.4** | **1.5** |
| Conway et al. (2017) | Yes | Yes | Yes | Yes (In-depth interviews allowed for 'yarning', a culturally appropriate data collection method) | Yes | Yes (Findings are supported by rich verbatim quotes and member-checking) | Yes (Strong coherence between the qualitative cases and the thematic analysis results) |
| Cook & Wills (2012) | Yes | Yes | Yes | Yes | Yes | Yes (Findings were substantiated by verbatim quotes across different employment sectors) | Yes (Strong coherence between the exploratory aims and the qualitative methodology) |
| Coritsidis et al. (2025) | Yes | Yes | Yes | Yes | Yes | Yes (Findings are supported by rich verbatim quotes from the 21 participants) | Yes (Clear coherence between the participatory design and the resulting policy recommendations) |
| D’Arcy et al. (2019) | Yes | Yes | Yes | Yes (Triangulation of online activity logs and interviews provided sufficient data depth) | Yes (A hybrid deductive-inductive thematic analysis was logically applied) | Yes (Interpretations are supported by verbatim participant quotes) | Yes (Strong coherence between the socio-ecological framework and the identified subthemes) |
| Fuller et al. (2024) | Yes | Yes | Yes | Yes (Interviews with 14 key informants across two jurisdictions provided necessary depth) | Yes (Thematic analysis mapped to Proctor’s framework was logically performed) | Yes (Findings on 'gatekeeping' and 'role confusion' are supported by leader quotes) | Yes (Clear coherence between the quality improvement focus and the implementation findings) |
| George et al. (2020) | Yes | Yes | Yes | Yes (Interviews with leaders from all 16 state CCOs provided comprehensive coverage) | Yes (Themes regarding employment structures and financial barriers were logically derived) | Yes (Findings are substantiated by quotes from clinical, quality, and community health leaders) | Yes (Strong coherence between the multidisciplinary research team and the analysis) |
| George et al. (2025) | Yes | Yes | Yes | Yes | Yes (Rapid qualitative matrix analysis was logically applied to identify themes) | Yes (Findings on 'role confusion' and 'power dynamics' are supported by verbatim participant quotes) | Yes (Clear coherence between the CFIR framework and the implementation results) |
| Gunter et al. (2023) | Yes | Yes | Yes | Yes | No (Lack of detail on data collection and analysis methods impedes judging coherence and adequacy) | Yes (Findings are supported by detailed legislative excerpts and specific operational strategies) | Can’t tell (Lack of detail on data collection and analysis methods impedes judging coherence) |
| Haughton et al. (2020) | Yes | Yes | Yes | Yes (Interviews with 22 pastors and staff across diverse denominations provided necessary depth) | Yes (Thematic analysis mapped to CFIR and ERIC strategies was logically performed) | Yes (Findings on 'pastor self-efficacy' and 'mission alignment' are supported by verbatim quotes) | Yes (Clear coherence between the implementation science framework and the recommendations) |

| **Study** | **S1** | **S2** | **1.1** | **1.2** | **1.3** | **1.4** | **1.5** |
| --- | --- | --- | --- | --- | --- | --- | --- |
| Hernandez-Salinas et al. (2023) | Yes | Yes | Yes (Qualitative phenomenology/narrative is appropriate for documenting lived experiences and dual roles) | Yes (Five focus groups with 27 experienced CHWs provided sufficient depth) | Yes (Thematic analysis in the original language by bilingual coders ensured logical derivation) | Yes (Findings on 'misinformation' and 'burnout' are substantiated by verbatim Spanish quotes and translations) | Yes (Strong coherence between ecological systems theory and the multi-level thematic results) |
| Hood et al. (2024) | Yes | Yes | Yes | Yes | Yes (Themes were logically derived using the CFIR framework) | Yes (Findings were substantiated by verbatim quotes across stakeholder groups) | Yes (Clear coherence between the network partnership context and the thematic results) |
| Ingram et al. (2025) | Yes | Yes | Yes | Yes | Yes (Thematic analysis co-led by bilingual partners ensured logical derivation of themes) | Yes (Findings are well-substantiated by verbatim quotes from leadership, supervisors, and CHWs) | Yes (Clear coherence between the participatory CBPR methodology and the resulting thematic framework) |
| Johnson et al. (2022) | Yes | Yes | Yes | Yes | Yes | Yes (Findings are supported by detailed accounts of curriculum and training) | Yes (Strong coherence between the CBPR framework and the resulting lessons learned) |
| Koskan et al. (2013) | Yes | Yes | Yes | Yes | Yes (Themes regarding rationale, logistics, and reframing were logically derived via inductive coding) | Yes (Findings are supported by verbatim quotes from researchers, directors, and trainers) | Yes (Strong coherence between the practice-focused objective and the qualitative results) |
| Kuroda et al. (2024) | Yes | Yes | Yes | Yes | Yes | Yes (Findings are well-substantiated by verbatim quotes across four main themes) | Yes (Clear coherence between the social constructivist framework and the resulting thematic evidence) |
| Lapidos et al. (2021) | Yes | Yes | Yes | Yes | Yes | Yes (Findings are well-substantiated by verbatim quotes across all themes) | Yes (Clear coherence between the real-world practice objectives and the qualitative results) |
| Mason et al. (2011) | Yes | Yes | Yes | Yes | Yes | Yes (Findings regarding substantiated) | Yes (Clear coherence between goal and the policy lessons) |
| **Study** | **S1** | **S2** | **1.1** | **1.2** | **1.3** | **1.4** | **1.5** |
| McAlearney et al. (2020) | Yes | Yes | Yes | Yes | Yes | Yes (Findings are supported by verbatim quotes from staff, preceptors, and CHWs) | Yes (Strong coherence between the workforce development aim and the qualitative implementation themes) |
| McCarville et al. (2024) | Yes | Yes | Yes | Yes | Yes | Yes (Findings are supported by verbatim quotes across individual, team, and organizational levels) | Yes (Strong coherence between the human factors engineering framework and the resulting integration factors) |
| Menser et al. (2020) | Yes | Yes | Yes | Yes | Yes | Yes (Findings on 'safety concerns' and 'funding cuts' are substantiated by verbatim quotes) | Yes (Clear coherence between the implementation science framework and the resulting themes) |
| Morey et al. (2025) | Yes | Yes | Yes | Yes ( | Yes (Abductive thematic analysis was logically applied to identify cross-cutting themes) | Yes (Findings on 'systemic undervalue' and 'relational capital' are substantiated by verbatim quotes) | Yes (Clear coherence between the NIMHD framework and the resulting structural recommendations) |
| Obasanjo et al. (2024) | Yes | Yes | Yes | Yes | Yes | Yes (Findings on 'resilience' and 'trust' are substantiated by verbatim participant quotes) | Yes (Clear coherence between the research objective and the resulting themes) |
| Orpinas et al. (2020) | Yes | Yes | Yes | Yes | Yes (Thematic analysis results were logically derived via line-by-line coding) | Yes (Findings are supported by rich thematic descriptions and participant-derived strategies) | Yes (Clear coherence between the intersectionality/resilience framework and the qualitative results) |
| Palmer-Wackerly et al. (2019) | Yes | Yes | Yes | Yes | Yes | Yes (Findings are well-substantiated by verbatim participant quotes across three ecological levels) | Yes (Clear coherence between the CCA theoretical framework and the resulting themes) |
| Payne et al. (2017) | Yes | Yes | Yes | Yes | Yes | Yes (Findings are supported by direct quotes from project leaders, partners, and CHWs) | Yes (Clear coherence between the organizational perspective and the resulting integration themes) |

| **Study** | **S1** | **S2** | **1.1** | **1.2** | **1.3** | **1.4** | **1.5** |
| --- | --- | --- | --- | --- | --- | --- | --- |
| Perez et al. (2024) | Yes | Yes | Yes | Yes | Yes | Yes (Findings are supported by verbatim quotes from the CHW participants) | Yes (Clear coherence between the social work theoretical framework and the resulting themes) |
| Polletta et al. (2021) | Yes | Yes | Yes | Yes | Yes (Thematic analysis via grounded theory | Yes (Findings are well-substantiated by verbatim quotes across all staff categories) | Yes (Clear coherence between the CCM/CHW framework and the resulting thematic evidence) |
| Rahman et al. (2021) | Yes | Yes | Yes | Yes |  | Yes (Findings are well-substantiated by verbatim quotes) | Yes (Clear coherence between the study's aim and the resulting themes) |
| Rodriguez Espinosa et al. (2024) | Yes | Yes | Yes | Yes (Triangulation of focus groups, survey data, and meeting notes) | Yes | Yes (Findings are supported by qualitative accounts from the 75 community partners) | Yes (Clear coherence between the community-centred purpose and the thematic results) |
| Rogers et al. (2018) | Yes | Yes | Yes | Yes | Yes | Yes (Findings are supported by rich verbatim quotes and summaries of informant perspectives) | Yes (Clear coherence between the research purpose and the resulting themes of clinic culture/leadership) |
| Rojas et al. (2015) | Yes | Yes | Yes | Yes | Yes | Yes | Yes (Clear coherence between the research objective and the resulting thematic evidence) |
| Sainz et al. (2024) | Yes | Yes | Yes | Yes | Yes | Yes (Findings are supported by rich verbatim quotes illustrating themes of structural barriers) | Yes (Clear coherence between the study's aim and the resulting thematic evidence) |
| Schriger et al. (2024) | Yes | Yes | Yes | Yes (39 interviews provided sufficient depth) | Yes (Rapid qualitative analytic used to categorize themes by CFIR domains) | Yes (Findings are well-substantiated by verbatim quotes from all constituent groups) | Yes (Clear coherence between the CFIR framework and the resulting barriers/facilitators) |
| Sherman et al. (2017) | Yes | Yes | Yes | Yes | Yes | Yes (Findings are supported by illustrative quotes from both CHWs and supervisors) | Yes (Clear coherence between the study's focus on workplace settings and the resulting themes) |
| **Study** | **S1** | **S2** | **1.1** | **1.2** | **1.3** | **1.4** | **1.5** |
| Smithwick et al. (2023) | Yes | Yes | Yes | Yes (Data from multiple levels provided extensive depth) | Yes | Yes (Findings well-substantiated by verbatim quotes) | Yes (Clear coherence between the study's aim and the resulting career frameworks) |
| Stupplebeen et al. (2019) | Yes | Yes | Yes | Yes | Yes | Yes (Findings are supported by verbatim quotes regarding isolation, burnout, and funding) | Yes (Clear coherence between the evaluation goal and the resulting thematic evidence) |
| Toney et al. (2022) | Yes | Yes | Yes | Yes | Yes | Yes (Themes like 'Financial Hardships' and 'Supervisor Support' are well-supported by verbatim quotes) | Yes (Clear coherence between the ecological theoretical framework and the resulting thematic evidence) |
| Tredinnick-Rowe et al. (2024) | Yes | Yes | Yes | Yes | Yes | Yes (Findings on "door-knocking" challenges and NHS integration are well-supported by participant quotes) | Yes (Clear coherence between the pilot's aim to describe spread and the resulting implementation guide) |
| Trejo et al. (2013) | Yes | Yes | Yes | Yes | Yes | Yes (Findings are well-substantiated by verbatim quotes regarding teaching efficacy and family support) | Yes (Clear coherence between the study's aim and the resulting themes of self-efficacy and empowerment) |
| Washburn et al. (2022) | Yes | Yes | Yes | Yes | Yes (Deductive and inductive thematic analysis) | Yes (Findings are supported by rich verbatim quotes illustrating the three stages of integration) | Yes (Clear coherence between the research aim and the resulting stages of the relationship framework) |
| Wennerstrom et al. (2024) | Yes | Yes | Yes | Yes | Yes | Yes (Findings are well-substantiated by verbatim quotes) | Yes (Clear coherence between the quality improvement objective and the resulting lessons learned) |
| Wilkinson et al. (2016) | Yes | Yes | Yes | Yes | Yes (Thematic findings are logically derived from the participatory case study analysis) | Yes (Findings are supported by detailed case evidence) | Yes (Clear coherence between the goal of describing a "partnership model" and the resulting policy lessons) |

| **Study** | **S1** | **S2** | **1.1** | **1.2** | **1.3** | **1.4** | **1.5** |
| --- | --- | --- | --- | --- | --- | --- | --- |
| Williamson et al. (2022) | Yes | Yes | Yes | Yes | Yes | Yes (Findings are well-supported by verbatim quotes regarding trust, navigation, and system constraints) | Yes (Clear coherence between the pilot evaluation goals and the resulting thematic evidence) |

**Quantitative studies**

| **Study** | **S1** | **S2** | **4.1** | **4.2** | **4.3** | **4.4** | **4.5** |
| --- | --- | --- | --- | --- | --- | --- | --- |
| Kirkland et al. (2024) | Yes | Yes | Yes | Yes | Yes (The 9-item organisational support variable demonstrated high internal consistency) | Yes (The use of balanced repeated replication survey estimations addressed potential sampling bias) | Yes (Stepwise logistic regression and postestimation margins appropriate) |
| Rohan et al. (2024) | Yes | Yes | Yes (The sample is representative of U.S. PCPs) | Yes (Measurements utilised a nationally endorsed framework, C3, for defining CHW roles) | Yes (Nonresponse bias is low with a 65.8% response rate; data excludes outliers like paediatricians for focus) | Yes (Risk of bias is low; validated online panels and standardised definitions were used) | Yes (Descriptive and chi-square analyses appropriate) |
| Scanlon et al. (2025) | Yes | Yes | Yes (The sample is representative of Indiana CHWs) | Yes (Measurements used validated tools adapted from MiCHWA and the C3 core consensus project) | Yes (The 282 eligible respondents represent a significant portion of CHWs in state) | Yes (Risk of bias is low; anonymous data collection) | Yes (Statistical analyses, including bivariate correlations for salary, were appropriate) |

**Mixed methods studies**

| **Study** | **S1** | **S2** | **5.1** | **5.2** | **5.3** | **5.4** | **5.5** |
| --- | --- | --- | --- | --- | --- | --- | --- |
| Acharya et al. (2024) | Yes | Yes |  | Yes (Integration achieved by using CHW pilot feedback to refine the final "production" database) | Yes | Yes (Addressed the interoperability limitation while highlighting high user satisfaction) | Yes (QUAN: Friedman test for feature ranking; QUAL: Inductive content analysis) |
| Adinkrah et al. (2024) | Yes | Yes | Yes (Rationale provided: to capture both measurable impact and nuanced lived experiences) | Yes (Integration occurred at the interpretation stage using the CFIR framework) | Yes (Discussion synthesises how qualitative adaptations explained quantitative completion rates) | No (Focus on Christian FBOs only; short-term nature restricts assessment) | Yes (QUAL: Appropriate CBPR approach; QUAN: Sampling strategy relevant to target population) |
| **Study** | **S1** | **S2** | **5.1** | **5.2** | **5.3** | **5.4** | **5.5** |
| Allen et al. (2015) | Yes | Yes | Yes (Rationale provided) | Yes (Triangulation used qualitative data to explain quantitative correlations) | Yes (Interpretations reflect the value added by combining both methods) | Yes (No significant divergences found between the survey and interview results) | Yes (QUAN: Validated pilot-tested survey; QUAL: Verbatim transcripts and thematic coding) |
| Casillas et al. (2022) | Yes | Yes | Yes (Rationale clear) | Yes (Integration achieved using survey data to provide context for the metrics reported) | Yes | No (Divergences not addressed) | Partial (Total invited N not reported; potential reporting bias in reports) |
| Foo et al. (2024) | Yes | Yes | Yes (Rationale clear) | Yes (Integration achieved by using qualitative interviews to explain the quantitative dose-response associations) | Yes (Meta-inferences connect specific CHW activities like 'alliance building' to successful cessation outcomes) | Yes (Addressed divergences such as mixed success in care coordination despite high participant engagement) | Yes (QUAN: Logistic regression on trial data; QUAL: Thematic analysis using CFIR framework with intercoder reliability) |
| Gore et al. (2019) | Yes | Yes | Yes (The rationale is clear) | Yes | Yes (The authors successfully link quantitative self-efficacy scores with qualitative concerns about training and added workload) | No (does not explicitly highlight or reconcile contradictions between the high survey interest and uncertainty expressed in interviews) | Yes/Partial (QUAN: High response rate (96%), but small sample (n=54) limits generalisability. QUAL: Rigorous thematic analysis using CFIR framework) |
| Grice-Jackson et al. (2024) | Yes | Yes | Yes (Rationale clear) | Yes (Integration achieved by using qualitative findings to explain the reach and adoption metrics) | Yes (CHW empowerment linked to the significant quantitative risk reduction observed) | Yes (Addressed divergences, such as the difficulty in recruiting men despite gender-neutral social media outreach) | Yes (QUAL: Framework analysis with stakeholder triangulation; QUAN: Validated CVD risk tools and non-parametric tests) |
| King et al. (2017) | Yes | Yes | Yes | Yes (Data mixing occurred during interpretation to expand on reported training successes) | Yes (connects strengthened PHN-CHR alliances to improved health coaching) | No (notes variability in integration across service units, but doesn’t detail how contradictions reconciled) | Yes (QUAL: Inductive thematic analysis; QUAN: Descriptive statistics and Chi-square tests) |
| Kirimi (2024) | Yes | Yes | Yes | No (presents three separate manuscripts, the integration isn’t transparent) | Yes | Yes (Adequately addresses the tension between CHWs viewing the role as a calling vs material pressures) | Yes (QUAN: Descriptive statistics/rankings of 150 CHWs; QUAL: Thematic analysis of 7 focus groups) |
| Mathers et al. (2014) | Yes | Yes | Yes | Yes (Integration achieved by using qualitative accounts to explain why patterns changed over time) | Yes (links the medicalisation of the role to the requirements of NHS performance management) | Yes (Addressed the divergence between the original community-facing policy vision and the eventual changes) | Yes (QUAL: Framework thematic analysis; QUAN: Descriptive statistics of throughput and referral sources) |
| **Study** | **S1** | **S2** | **5.1** | **5.2** | **5.3** | **5.4** | **5.5** |
| McCutcheon et al. (2025) | Yes | Yes | Yes | Yes (Integration achieved by using qualitative focus groups to explain differences in patient populations seen) | Yes (connects the invisible status of CHWs to the high referral volume found in data) | Yes (Divergences between models explicitly addressed) | Yes (QUAL: Reflexive thematic analysis; QUAN: Bivariate analysis of EHR data) |
| McCutcheon et al. (2025) | Yes | Yes | Yes | Yes (Integration achieved by using qualitative focus groups to explain differences in patient populations seen in the EHR) | Yes | Yes (Divergences between models explicitly addressed) | Yes (QUAL: Reflexive thematic analysis; QUAN: Bivariate analysis of EHR data) |
| Paulson et al. (2021) | Yes | Yes | Yes | Yes (Integration achieved by using qualitative insights to explain variations in quantitative referral types) | Yes | Yes (Addressed divergences, such as provider scepticism vs programme appreciation) | Yes (QUAL: Thematic analysis; QUAN: Descriptive statistics) |
| Porteny et al. (2020) | Yes | Yes | Yes | Yes (Integration achieved by using qualitative findings to elaborate on quantitative survey results) | Yes | Yes (Divergences between high program value and high burnout/turnover were explicitly addressed) | Yes (QUAN: Median/IQR descriptive stats; QUAL: Thematic axial coding) |
| Rodriguez et al. (2022) | Yes | Yes | Yes | Yes (Integration achieved via data consolidation and cross-tabulation of topics) | Yes | Yes (Addressed unique findings from each source) | Yes (QUAN: Descriptive statistics; QUAL: Inductive/deductive thematic analysis) |
| Sharma et al. (2025) | Yes | Yes | Yes | Yes | Yes | Yes (Addressed the tension between CHWs' high value-add and their low organisational job security) | Yes (QUAN: Descriptive statistics of 21 orgs; QUAL: Inductive thematic analysis) |
| Sursely et al. (2025) | Yes | Yes | Yes | Yes | Yes (links role ambiguity and funding instability to workforce expansion barriers) | Yes (Addressed divergent perceptions of pay and training needs between the two groups) | Yes (QUAN: Descriptive statistics; QUAL: Thematic analysis) |
| Van der Wees et al. (2014) | Yes | Yes | Yes | Yes (Integration achieved by using surveys to assess baseline readiness and interviews to explain implementation variability) | Yes | Yes (Addressed why MAs felt crowded out by clinical tasks while CHWs built specialised expertise) | Yes (QUAN: Descriptive statistics/t-tests; QUAL: Directed content analysis) |

**Additional Resource 3: GRADE‐CERQual assessment**

| **Summary of review findings** | **Methodological limitations** | **Coherence** | **Adequacy** | **Relevance** | **Confidence in the evidence** | **Explanation of GRADE‐CERQual assessment** |  |
| --- | --- | --- | --- | --- | --- | --- | --- |
| **Outer setting factors** | | | | | | | |
| **Government / policy factors** | | | | | | | |
| [41-44, 46, 91, 52, 54, 94, 107, 59-61, 27, 63, 108, 135, 68, 100, 110, 79, 111, 102, 103, 82, 104, 87, 109] | Minor concerns: Issues combining data types, but does not impact conclusions drawn | Moderate concerns: Findings are interpretive, with some transformation regarding buy-in and ignorance of other intersecting / unknown factors. But are reasonable and grounded in data | Moderate concerns: data from just one study, but richly described | Minor concerns: Partially relevant to the RQ; indicative of the complexity of implementing CHW programmes via policy paper | Moderate confidence | Moderate concerns regarding coherence and adequacy  Minor concerns regarding method and relevance |  |
| **Certification** | | | | | | | |
| [42, 46, 106, 48, 52, 54, 94, 60, 61, 95, 108, 65, 67, 71, 76, 101, 111, 102, 103, 82, 104, 83, 87, 109] | No concerns | Minor concerns: finding is descriptive but may lack some nuance regarding the complex issue | Moderate concerns: studies were very familiar with certification issues, but only a small number covered the topic | No concerns: All papers relevant to the RQ; indicative of the complexity of implementing CHW programmes in the US context | Moderate confidence | Moderate concerns regarding adequacy  Minor concerns regarding coherence |  |
| **COVID-19** | | | | | | | |
| [89, 42-47, 91, 52, 94, 58-61, 96, 63, 68, 69, 72, 100, 110, 79, 111, 80, 102, 104, 87] | No concerns | Minor concerns: the finding is broad and descriptive | Minor concerns: the theme was referenced in most studied published since 2021 | No concerns: the finding is relevant to the RQ | High confidence | Minor concerns regarding coherence and adequacy |  |
| **Summary of review findings** | **Methodological limitations** | **Coherence** | **Adequacy** | **Relevance** | **Confidence in the evidence** | **Explanation of GRADE‐CERQual assessment** |  |
| **Health system factors** | | | | | | | |
| **Funding and sustainability** | | | | | | | |
| [41-44, 46, 106, 47, 91, 52, 54-56, 94, 107, 57, 58, 60, 61, 96, 27, 62, 64, 108, 135, 98, 67, 68, 99, 76, 100, 77, 110, 79, 111, 80, 102, 81, 103, 82, 104, 84, 87, 88] | No concerns | Minor concerns: the finding is broad and descriptive | Minor concerns: Data are from a range of studies surveying programme leaders; some are very rich about the concerns, though lacking in contextual detail | No concerns: Addresses a system level aspect of the RQ; funding mechanisms not discussed in detail | High confidence | Minor concerns regarding coherence, adequacy |  |
| **Organisational readiness** | | | | | | | |
| [89, 42-44, 91, 52, 54, 55, 107, 57, 59-61, 95, 63, 135, 66, 98, 68, 72, 100, 77, 110, 80, 102, 103, 84, 109] | No concerns | Moderate concerns: the finding is descriptive, but lacks nuance towards the complexities of organisational culture (which were not explored in the studies either) | Moderate concerns: the studies were robust and the participants strategically relevant, but this theme was treated superficially within the studies | No concerns: culture and readiness are known implementation determinants | Moderate confidence | Moderate concerns regarding coherence and adequacy |  |
| **Intervention factors** | | | | | | | |
| **Intervention design** | | | | | | | |
| [41-44, 46, 106, 48, 91, 50, 53, 92, 54, 55, 94, 57, 59, 61, 95, 96, 108, 68, 100, 111, 80, 102, 84, 85, 87, 109, 88] | No concerns | Moderate concerns. the finding is descriptive, but lacks nuance towards the complexities and variety of innovative practices involving CHWs | Moderate concerns: a small number of studies were represented in the finding, each describing interventions in quite different settings | No concerns: the evidence is relevant to the RQ | Moderate confidence | Moderate concerns regarding coherence and adequacy |  |
| **Summary of review findings** | **Methodological limitations** | **Coherence** | **Adequacy** | **Relevance** | **Confidence in the evidence** | **Explanation of GRADE‐CERQual assessment** |  |
| **Recruitment** | | | | | | | |
| [89, 42, 106, 47-49, 91, 54, 94, 57, 61, 96, 27, 135, 66-68, 72, 73, 76, 101, 110, 79, 111, 103, 104, 87] | No concerns | No concerns: finding is descriptive | Moderate concerns: data from a range of studies; recruitment was a minor factor in each and description was not especially rich | No concerns: studies in the finding had varied levels of relevance to the overall RQ; their evidence on recruitment was complementary | Moderate confidence | Moderate concerns regarding adequacy |  |
| **Training** | | | | | | | |
| [41, 89, 42, 136, 44, 46, 106, 48, 49, 91, 52, 92, 54, 55, 93, 94, 57, 59-61, 95, 96, 27, 62, 63, 108, 135, 65, 66, 98, 67, 68, 71, 73, 99, 76, 100, 101, 111, 80, 102, 81, 103, 82, 104, 84, 87, 109] | No concerns | Minor concerns: the finding is descriptive and fairly detailed; some nuance around contrasting approaches is lost | Minor concerns: a large number of studies and perspectives; some aspects treated briefly | No concerns: the evidence is relevant to the RQ | High confidence | Minor concerns regarding coherence and adequacy |  |
| **Incentives** | | | | | | | |
| [42, 44, 46, 106, 49, 52, 54, 59, 60, 96, 27, 62, 108, 68, 99, 76, 100, 79, 111, 102, 103, 82, 104, 109] | No concerns | Minor concerns. A descriptive finding | Moderate concerns: The issue of incentives appears a relatively minor one in the few studies included | No concerns: the issue is partially relevant to the RQ, though intersecting with other factors like finance and supervision | Moderate confidence | Moderate concerns regarding adequacy.  Minor concerns regarding coherence |  |
| **Supervision** | | | | | | | |
| [41, 89, 42, 136, 44, 46, 106, 48, 91, 52, 53, 92, 54, 55, 94, 107, 59-61, 95, 96, 62, 63, 67, 73, 99, 76, 100, 101, 79, 111, 80, 102, 103, 82, 104, 83-85, 105, 87] | Minor concerns: methodological weaknesses for Gore and Allen | Moderate concerns: the finding is descriptive; it covers principle aspects of the data, but ignores some as the data are rich | Minor concerns: A large number of studies contributed with some specifically looking into this topic | No concerns: partial relevance; many of the included studies have direct relevance to the RQ | Moderate confidence | Moderate concerns regarding coherence.  Minor concerns regarding methodology and adequacy |  |
| **Summary of review findings** | **Methodological limitations** | **Coherence** | **Adequacy** | **Relevance** | **Confidence in the evidence** | **Explanation of GRADE‐CERQual assessment** |  |
| **Communication and integration** | | | | | | | |
| [41, 89, 42, 136, 43, 44, 46, 106, 48, 52, 53, 92, 54-56, 93, 94, 59, 60, 95, 96, 63, 108, 135, 65, 98, 68, 72, 73, 75, 76, 100, 77, 110, 78-80, 102, 81, 103, 82, 104, 83, 84, 105, 86, 87, 109, 88] | No concerns | Moderate concerns: the finding is descriptive; it lacks nuance regarding CHW roles and responsibilities | Minor concerns: a large evidence base; many studies prioritised evidence around communication and understanding | No concerns | High confidence | Moderate concerns regarding coherence  Minor concerns regarding adequacy |  |
| **Quality assurance and evaluation** | | | | | | | |
| [41, 89, 44, 46, 106, 49, 56, 61, 95, 96, 62, 63, 108, 135, 73, 100, 77, 79, 111, 102, 84, 105, 87] | No concerns | Minor concerns: the finding is broad and descriptive; some nuance is lost by focussing on CBOs | Moderate concerns: a number of studies touch on the topic and evidence is provided by strategic leaders: it’s a complex topic, however, and the evidence is thin in some areas | No concerns | Moderate confidence | Moderate concerns regarding adequacy  Minor concerns regarding coherence |  |
| **IT and data systems** | | | | | | | |
| [41, 89, 136, 44, 106, 91, 54, 55, 94, 107, 59, 95, 96, 27, 135, 65, 98, 67, 69-73, 76, 100, 111, 102, 81, 84, 105, 87] | No concerns | Moderate concerns: the finding is descriptive but lacks some of the nuance of the complex topic | Moderate concerns: some studies go into detail about the complex topic, but it’s treated superficially elsewhere | Minor concerns: partial relevance to the RQ | Moderate confidence | Moderate concerns regarding coherence and adequacy  Minor concerns regarding relevance |  |
| **Summary of review findings** | **Methodological limitations** | **Coherence** | **Adequacy** | **Relevance** | **Confidence in the evidence** | **Explanation of GRADE‐CERQual assessment** |  |
| **Other resources** | | | | | | | |
| [89, 42, 44, 46, 106, 47, 49, 91, 55-57, 59, 61, 95, 96, 27, 62, 63, 108, 65, 66, 98, 68, 69, 73, 75, 99-101, 79, 111, 81, 82, 104] | No concerns | No concerns: the finding is broad and descriptive | Moderate concerns: the finding and the data touch on different aspects of the topic; in most examples the issues as address briefly | Minor concerns: partial relevance to the RQ; | Moderate confidence | Moderate concerns regarding adequacy  Minor concerns regarding relevance |  |
| **Engaging clients** | | | | | | | |
| [41, 89, 42, 136, 44-46, 106, 48, 91, 50, 51, 53, 92, 54, 55, 94, 57-61, 95, 96, 63, 64, 108, 98, 68, 70-72, 74, 100, 110, 79, 111, 80, 102, 81, 103, 82, 104, 84-87] | No concerns | Minor concerns: the finding is descriptive; some nuance is absent | Moderate concerns: the issues is touched on in a number of studies; they did not tend to provide detailed examples | No concerns: partial relevance to the RQ | Moderate confidence | Moderate concerns regarding adequacy  Minor concerns regarding coherence |  |
| **Engaging partner organisations** | | | | | | | |
| [41, 89, 42-46, 106, 91, 53, 92, 54, 94, 107, 57, 59, 61, 95, 96, 62, 63, 108, 65, 66, 98, 68, 72, 74, 75, 100, 79, 111, 102, 81, 103, 82, 104, 84, 105, 87, 109, 88] | No concerns | Moderate concerns: a broad and descriptive finding; it lacks nuance about these complex relationships | Serious concerns: the issue is treated superficially in some studies; the views of external organisations are represented indirectly by internal participants | No concerns: partial relevance to the RQ | Moderate confidence | Serious concerns regarding adequacy.  Moderate concerns regarding coherence |  |

| **Summary of review findings** | **Methodological limitations** | **Coherence** | **Adequacy** | **Relevance** | **Confidence in the evidence** | **Explanation of GRADE‐CERQual assessment** |  |
| --- | --- | --- | --- | --- | --- | --- | --- |
| **Individual level factors** | | | | | | | |
| **CHW motivation and self-efficacy** | | | | | | | |
| [41, 89, 42, 136, 43, 44, 46, 106, 48-50, 52, 53, 92, 54, 55, 94, 58-61, 95, 96, 62, 63, 108, 65, 98, 68, 69, 71, 100, 101, 79, 111, 102, 103, 82, 104, 83-85, 87] | No concerns | Moderate concerns: the finding is broad and descriptive; it perhaps lacks some of the data’s nuance | Moderate concerns: some studies provide rich evidence of CHW motivation; in others, data are reported indirectly by service leaders | No concerns: partial relevance to the RQ | Moderate confidence | Moderate concerns regarding adequacy and coherence |  |
| **Service users** | | | | | | | |
| [89, 106, 48, 91, 50-53, 92, 54, 55, 94, 107, 59-61, 63, 64, 108, 135, 65, 66, 70, 72, 100, 101, 79-81, 104, 83, 84, 88] | No concerns | Minor concerns: the finding is broad and descriptive; it lacks some of the nuance of the data | Moderate concerns: most studies did not directly assess service user perspectives | Minor concerns: The RQ asks what influences CHW implementation: the client perspective is directly relevant | Moderate confidence | Moderate concerns regarding adequacy.  Minor concerns regarding coherence and relevance |  |
